# MicroRNA Profiling of Epstein-Barr Virus-Associated NK/T-Cell Lymphomas by Deep Sequencing

**DOI:** 10.1371/journal.pone.0042193

**Published:** 2012-08-03

**Authors:** Natalie Motsch, Julia Alles, Jochen Imig, Jiayun Zhu, Stephanie Barth, Tanja Reineke, Marianne Tinguely, Sergio Cogliatti, Anne Dueck, Gunter Meister, Christoph Renner, Friedrich A. Grässer

**Affiliations:** 1 Institute of Microbiology and Hygiene, Department of Virology, University Hospital of Saarland University, Homburg/Saar, Germany; 2 Laboratory of RNA Biology, Center for Integrated Protein Sciences Munich (CIPSM), Max Planck Institute of Biochemistry, Martinsried, Germany; 3 Institute of Surgical Pathology, University Hospital Zurich, Zurich, Switzerland; 4 Institute of Pathology, Kantonsspital St Gallen, St Gallen, Switzerland; 5 Biochemistry I, Regensburg University, Regensburg, Germany; 6 Division of Oncology, Department of Internal Medicine-Oncology, University Hospital Zurich, Zurich, Switzerland; The University of Hong Kong, China

## Abstract

The Epstein-Barr virus (EBV) is an oncogenic human Herpes virus involved in the pathogenesis of nasal NK/T-cell lymphoma. EBV encodes microRNAs (miRNAs) and induces changes in the host cellular miRNA profile. MiRNAs are short non-coding RNAs of about 19–25 nt length that regulate gene expression by post-transcriptional mechanisms and are frequently deregulated in human malignancies including cancer. The microRNA profiles of EBV-positive NK/T-cell lymphoma, non-infected T-cell lymphoma and normal thymus were established by deep sequencing of small RNA libraries. The comparison of the EBV-positive NK/T-cell vs. EBV-negative T-cell lymphoma revealed 15 up- und 16 down-regulated miRNAs. In contrast, the majority of miRNAs was repressed in the lymphomas compared to normal tissue. We also identified 10 novel miRNAs from known precursors and two so far unknown miRNAs. The sequencing results were confirmed for selected miRNAs by quantitative Real-Time PCR (qRT-PCR). We show that the proinflammatory cytokine interleukin 1 alpha (IL1A) is a target for miR-142-3p and the oncogenic BCL6 for miR-205. MiR-142-3p is down-regulated in the EBV-positive vs. EBV-negative lymphomas. MiR-205 was undetectable in EBV-negative lymphoma and strongly down-regulated in EBV-positive NK/T-cell lymphoma as compared to thymus. The targets were confirmed by reporter assays and by down-regulation of the proteins by ectopic expression of the cognate miRNAs. Taken together, our findings demonstrate the relevance of deregulated miRNAs for the post-transcriptional gene regulation in nasal NK/T-cell lymphomas.

## Introduction

The Epstein-Barr Virus (EBV) is an oncogenic human Herpes virus that is involved in the pathogenesis of nasopharyngeal carcinoma (NPC), stomach carcinoma and various tumours of B- and T-cell origin such as Burkitt’s and Hodgkin’s lymphoma, diffuse large B-cell lymphoma (DLBCL) and nasal NK/T-cell lymphoma (for review, see [1]). Its oncogenic property is highlighted by the ability of EBV to growth-transform B-lymphocytes; these so-called lymphoblastoid cell lines (LCL’s) are the *in vitro* correlate of B-cell lymphoproliferative disorders that often arise under immunosuppression. In the various EBV-associated tumour entities, the virus expresses different sets of transformation-associated proteins as well as non-coding RNAs [2]. These include the so-called EBER-RNAs, a snoRNA [3] and a set of 25 miRNAs [4], [5], [6]. MiRNAs are short, 19–25 nt RNAs with partial homology to sequences in their target mRNAs. MiRNA genes are transcribed and processed in the nucleus, then exported to the cytoplasm where they are further processed and ultimately bound in most cases to the 3′ untranslated region (UTR) of their target mRNA by the RNA-induced-silencing-complex (RISC). MiRNAs were also reported to bind to their targets via 5′UTR or open reading frame [7], [8]. Association with a target mRNA results in either translational repression or mRNA degradation ultimately leading to reduced protein synthesis (for review, see [9]). EBV not only expresses its own set of miRNAs but also has a profound impact on the cellular miRNA profile in that the overall level of cellular miRNAs appears to be down-regulated in EBV-infected cells [10] and that the viral infection changes the levels of specific miRNAs. For instance, various cellular miRNAs are up- or down-regulated in NPC when compared to non-infected tissue [11]. Among the EBV-associated tumours, NPC and nasal NK/T-cell lymphoma are the two entities that are virtually always infected with EBV. NK/T-cell lymphomas are mainly found in South-east Asia where they constitute about 3–9% of all malignant lymphoma (Reviewed in [1]). The tumours mainly arise in the nasal region but also in extranodal areas of the gastro-intestinal tract, the skin, the liver or the spleen [12]. The tumours grow very aggressively and are characterized by large necrotic areas probably due to the secretion of large amounts of proteinases [13]. Therefore, only small amounts of tumour tissue are available. We nevertheless set out to determine the miRNA profiles of nasal NK/T-cell lymphoma in comparison to non-EBV-infected T-cell lymphoma using thymus as a non-transformed control tissue, by utilizing the deep sequencing as a powerful tool. In addition to establishing the miRNA profiles, we identified targets of the deregulated cellular miRNAs.

## Results

### Analysis of the Small RNA Libraries

**Figure 1:**
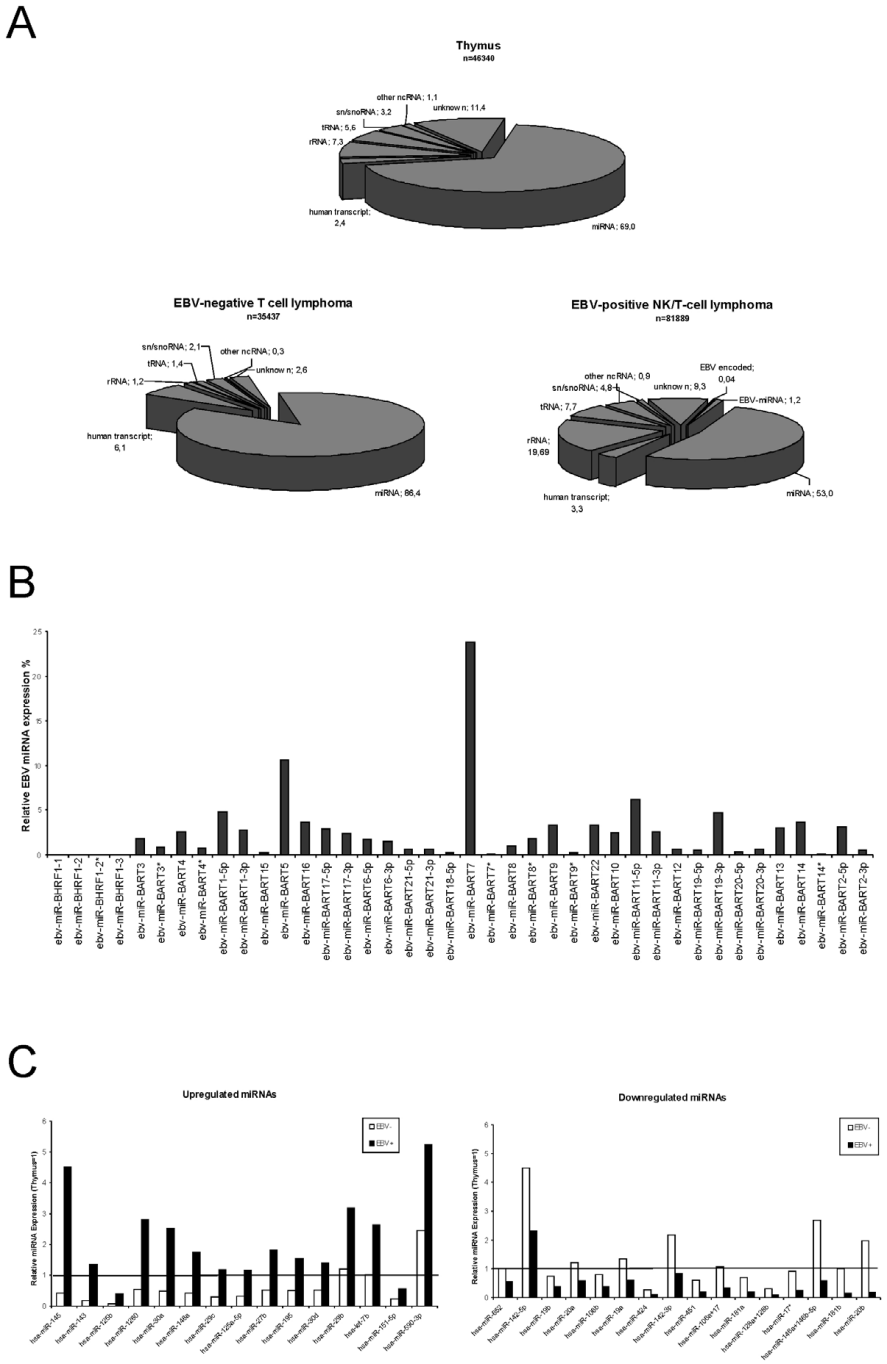
Expression of small ncRNAs in the analysed small RNA libraries. A: Proportions of ncRNAs and transcripts detected by deep sequencing of Thymus (total reads: 46340), EBV-negative T-cell (total reads: 35437), and EBV-associated NK/T-cell lymphoma (total reads: 81889). The relative abundance is calculated as a quotient of the absolute number of a single class of RNA and the total number of all detected sequences in the library. miRNA = microRNA; ncRNA = non-coding RNA; snRNA = small nuclear RNA; snoRNA = small nucleolar RNA; tRNA = transfer RNA; rRNA = ribsomal RNA B: Expression of EBV-encoded miRNAs in EBV-associated NK/T-cell lymphoma. Relative distribution of viral miRNAs represented as the percentage of the total EBV-encoded miRNA reads. No sequences were detected for miRNAs from the BHRF1-cluster C: Relative expression of deregulated miRNAs in EBV-positive NK/T-cell vs. EBV-negative T-cell lymphoma. The left diagram shows the up-regulated miRNAs whereas the right one represents the down-regulated miRNAs in EBV-associated NK/T-cell lymphomas. Presented is the relative miRNA expression as a percentage of the total miRNA reads in the small RNA library after setting the thymus expression to 1. Only miRNAs with an at least two-fold difference in expression and an expression of at least 0.1% are shown. The white bars represent the expression in EBV-negative T-cell lymphoma, the black ones the expression in the EBV-positive NK/T-cell lymphoma.

The miRNA profiles of EBV-positive nasal NK/T-cell lymphoma, EBV-negative T-cell lymphoma and non-transformed thymus tissue were established as previously described [11]. In brief, small RNA libraries were generated from pooled frozen tissues and analysed by 454 deep-sequencing. The distribution of reads obtained is schematically shown in Figure 1A. While the major part represented miRNAs, we also obtained a background of sequences derived from other RNAs such as rRNA, tRNA, sn/snoRNAs, other ncRNAs or mRNA transcripts. In the thymus small RNA library, 69% of the 46340 reads were identified as miRNA sequences, the small RNA library from EBV-negative lymphoma yielded 86% miRNA sequences from 35437 reads while EBV-positive NK/T-cell lymphoma library showed a reduced amount of 53% of miRNA-derived sequences among 81889 reads. Of the known human miRNAs, we identified 348 in the thymus library, 325 in the library from the EBV-positive but only 275 in the library of the EBV-negative T-cell lymphoma. The library of the EBV-positive lymphoma contained the additional 36 viral miRNAs as outlined in Figure 1B.

### Expression of Viral miRNAs in EBV-associated NK/T-cell Lymphomas

Of note, we did not observe any EBV-miRNAs encoded by the BHRF1 cluster (see Table S1) in accordance with previous results showing that these miRNAs are only found in cells that harbor the virus in a type III latency, such as a PTLD arising under immunosuppression [14] but not, for instance, in NPC or stomach carcinoma [11], [15]. In contrast to the situation in NPC, where the EBV miRNAs accounted for 5–19% of the total miRNAs, the viral miRNAs in NK/T-cell lymphoma represented only 2.3% of the total miRNAs. The five most expressed EBV-miRNAs BART7, BART5, BART11-5p, BART1-5p and BART19-3p accounted for ∼50% of the viral encoded and for ∼1% of the total miRNAs. To confirm the absence of the EBV-encoded miRNAs from the BHRF1 cluster, the EBV-positive NK/T-cell lines HANKI and NK-YS (latency type II) were analysed by northern blotting. As shown in supporting Figure S1 A, neither the EBV-positive cells nor the EBV-negative control cells NK92 or SUP-T1 expressed any of the BHRF1 miRNAs, while the EBV-positive B95.8 cells (which are in type III latency) clearly showed a signal.

### Deregulated miRNAs in EBV-positive NK/T-cell and EBV-negative T-cell Lymphomas Relative to Normal Tissue

We then compared the miRNA expression levels between the different small RNA libraries. For this analysis, we set a threshold of a 2-fold difference in expression and a representation of 0.1% in both small RNA libraries. First, we looked at the miRNA expression changes between the lymphomas and the normal tissue. In EBV-negative T-cell lymphoma we could detect an up-regulation for 14 miRNAs whereas 31 of the 45 deregulated miRNAs were repressed relative to thymus (supporting Table S2). For the EBV-positive lymphomas 18 miRNAs were induced while 28 of the 46 deregulated miRNAs showed a reduced expression (supporting Table S3). Interestingly, most of the up-regulated miRNAs in EBV-negative lymphoma were also induced in EBV-positive lymphoma compared to normal tissue. For example, miR-21 and miR-155 were among the four strongest induced miRNAs in both lymphomas. The miRNAs with a reduced expression in lymphoma relative to normal tissue only coincided partially e.g. for miR-218 and miR-200 family. Ten miRNAs that were present in the EBV-positive samples and thymus were not or almost un-detectable in the EBV-negative tumours (Table 1). Of note, miR-449a and miR-449b were also not present in thymus. The other nine miRNAs, mainly members of the miR-200 family, showed reduced levels or comparable expression in the EBV-positive lymphomas as compared to thymus.

**Table 1:** Induced miRNAs showing no or only weak expression in EBV-negative T-cell lymphoma.

miRNA	relative expression [%]		
	Thymus	EBV-	EBV+
hsa-miR-200a	0,26	0,00	0,09
hsa-miR-200b	2,17	0,00	1,18
hsa-miR-203	0,11	0,00	0,10
hsa-miR-204	0,12	0,00	0,07
hsa-miR-205	1,49	0,00	0,28
hsa-miR-429	0,15	0,00	0,07
hsa-miR-449a+449b	0,00	0,00	0,21
hsa-miR-141	0,36	0,01	0,21
hsa-miR-199b-5p	0,26	0,02	0,22
hsa-miR-200c	0,88	0,03	1,03

### Differentially Expressed miRNAs in EBV-positive NK/T-cell vs. EBV-negative T-cell Lymphomas

Furthermore, we compared the relative expression levels of the cellular miRNAs between EBV-positive and EBV–negative lymphomas. In the EBV-positive NK/T-cell lymphomas, 15 cellular miRNAs were up-regulated and 16 miRNAs were down-regulated relative to the EBV-negative ones. For the miRNAs shown in the diagram of Figure 1C, the level seen in thymus tissue was set to 1. The strongest relative induction was observed for miR-145 (11-fold), miR-143 (8-fold), and miR-125b (7-fold), while strongest reduction was determined for miR-20b (10-fold), miR-181b (8-fold), and miR-146a+b (5-fold). MiR-145, -1280, -30a, -29b, 590-3p and let-7b of the up-regulated miRNAs in the EBV-positive lymphomas were also induced relative to thymus. Otherwise, the expression of the most down-regulated miRNAs was reduced relative to the EBV-negative lymphoma as well as to normal tissue. Only miR-652, -20a, -19a, -142-3p and miR-146a+b-5p were down-regulated compared to EBV-negative lymphoma but showed no reduction in normal tissue. MiR-590-3p and miR-142-5p were the only miRNAs which were deregulated in EBV-positive lymphomas and at the same time induced in both types of lymphomas compared to normal tissue.

As pointed out, we compared only those miRNAs that were represented at least 0.1% in one of the libraries. The results for all miRNAs detected by sequencing in the three libraries are shown in Table S4.

### Identification of New Cellular miRNAs

The analysis of the three small RNA libraries revealed 10 novel star miRNA sequences derived from previously described miRNA precursors (Table 2). Of these, five were only detected in the small RNA library of the EBV-negative lymphoma, one was only present in the EBV-positive lymphoma and one was only detectable in thymus. We also detected three novel miRNA sequences in the small RNA analysis of the EBV-negative lymphoma shown in supporting Table S5. While this work was in progress, the miRNA denoted miR pot.42 in supporting Table S5 was deposited in miRBase as hsa-mir-3157 [16]. The table also lists the chromosomal location of the three sequences. The sequences were found in the human genome and also in other hominid apes as well. Also, the predicted precursor folded into a hairpin structure fitting all criteria for a true miRNA. We therefore PCR-amplified the three sequences including 150 nt of the 5′- and 3′-flanking regions; expression of the clones and subsequent northern blotting showed expression of precursors and the mature miRNAs of the expected size. However, northern blot analysis of the NK/T-cell lines did not yield any signal for the endogenous miRNA (supporting Figure S1B).

**Table 2:** New miRNAs from known precursors identified from different small RNA libraries.

name	sequence	genomic location	reads in libraries		
			Thymus	EBV-	EBV+
hsa-miR-874-5p	CGGCCCCACGCACCAGGGT	5q31.2	1		4
hsa-miR-1248-3p	TGCTAGCAGAGTACACACAAG	3q27.3			4
hsa-miR-153-2-5p	GTCATTTTTGTGATGTTGCAGC	7q36.3		4	
hsa-miR-98-3p	CTATACAACTTACTACTTTC	Xp11.22		1	
hsa-miR-382-3p	AATCATTCACGGACAACACTT	14q32.31		2	
hsa-miR-511-3p	AATGTGTAGCAAAAGACAG	10p12.33		10	
hsa-miR-1277-5p	TATATATATATATGTACGTAT	11q24		3	
hsa-miR-1185-3p	ATATACAGGGGGAGACTCTTAT	14q32.31	2		
has-miR-107-5p	AGCTTCTTTACAGTGTTGCCTTGT	10q23.31	3		1
hsa-miR-let-7c-3p	CTGTACAACCTTCTAGCTTTCC	21q21.2	2		

### Validation of miRNA Expression by Quantitative Real-time PCR

**Figure 2:**
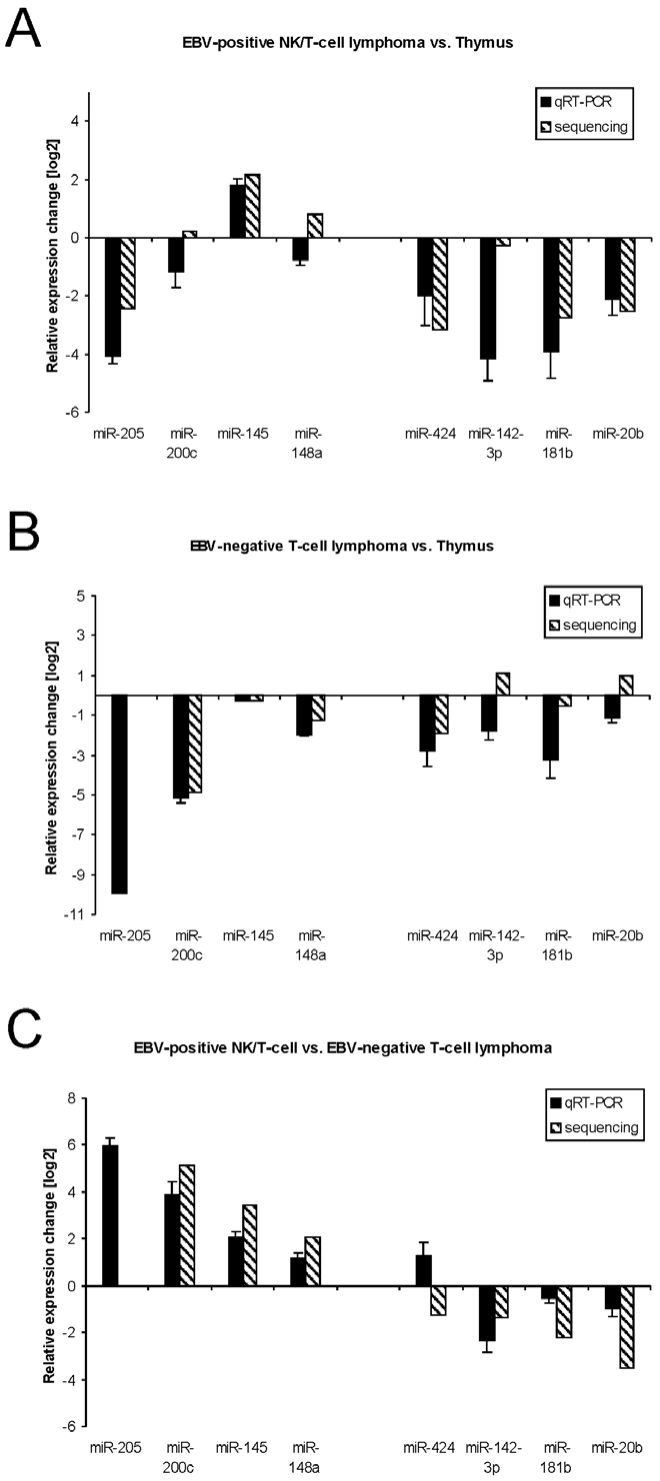
Validation of miRNA expression by quantitative Real-Time PCR. Total RNA out of the different samples used for sequencing was reverse transcribed and the expression of miRNAs was analysed in a light cycler by quantitative Real-Time PCR. The relative quantification was carried out by the 2^−ΔΔct^-method after measurement the amount of 5.8sRNA in each sample. Represented are the comparisons between (A) EBV-positive lymphomas and thymus, (B) EBV-negative lymphomas and thymus as well as (C) EBV-associated and EBV-negative lymphomas. The shaded bars represent the sequencing results and the filled bars the PCR-results. Bars above the x-axis show induced miRNAs and the bars below repressed miRNAs.

We next tried to confirm the sequencing data by qRT-PCR. As outlined above, the nasal NK/T-cell lymphoma contain large necrotic areas; thus only limited amounts of material were available. We therefore could only analyse the levels of 4 up- and 4 down-regulated miRNAs in the EBV-positive vs. -negative cases by PCR. We compared the expression of miR-205, -200c, -145, and -148a (up-regulated) as well as miR-424, -142-3p, -181b, and -20b (down-regulated). Figure 2 shows the results of the quantitative Real-Time PCR for the comparison between EBV-positive lymphoma and thymus (Figure 2A), EBV-negative and thymus (Figure 2B) as well as EBV-positive and -negative lymphoma (Figure 2C). For the lymphoma vs. thymus there were two miRNAs which showed a different expression change by PCR when compared to the sequencing results. For EBV-positive lymphoma vs. thymus, miR-200c and miR-148a were reduced whereas sequencing showed the same (miR-200c) or a slightly stronger (miR-148a) expression in thymus. For the EBV-negative lymphomas vs. thymus we found a reduced expression of miR-142-3p and miR-20b by PCR but a slight up-regulation in the lymphomas by sequencing.

When we compared the EBV-positive vs. EBV-negative lymphoma, all miRNAs but miR-424 were up- or down-regulated as measured by sequencing (see Figure 2C). By sequencing, miR-424 was down-regulated in EBV-positive vs. negative lymphomas and also down-regulated in both entities relative to thymus tissue but was found induced in EBV-positive vs. -negative lymphomas by PCR. The relative down-regulation of miR-424 for both lymphoma entities vs. thymus was confirmed, though. For all other miRNAs at least the same tendency of expression changes was detectable by PCR and by sequencing. Note that miR-205 was not detectable in the library of the EBV-negative lymphoma by sequencing; therefore, no relative values that compare the EBV-negative samples to the thymus and to the EBV-positive lymphomas can be displayed as log2. Nevertheless, the relative quantification of this miRNA by qRT-PCR confirmed the repression of miR-205 in EBV-negative lymphoma compared to thymus and the induction of the miRNA in EBV-positive ones.

We next analysed by qRT-PCR the relative expression levels of deregulated miRNAs in the NK/T-cell lines SNT6 and SNK16, one further case of nasal NK/T-cell lymphoma where frozen tissue was available and three additional cases of nasal NK/T-cell lymphoma where only paraffin-embedded material was available. These were either compared with primary thymus tissue or CD56+ cells isolated from healthy donors. It is assumed that CD56+/CD3+ cells are the precursor cells for EBV-positive NK/T-cell lymphoma [17]. When compared to primary thymus tissue, the tested miRNAs miR-142-3p, -145, -181b, -200c, -205 and –424 all were down-regulated in the tumor tissues and the cell lines tested, with the exception of miR-181b and miR-424 that essentially showed no change in the primary, frozen tissue of the NK/T-cell lymphoma. This is shown in supplementary Figure S3 and further confirms the original observations obtained by sequencing (see supporting Table S3). The comparison of CD56+ primary cells isolated from peripheral blood yielded essentially the same results with the exception of miR-205. Here, the tumor tissues expressed elevated levels of this miRNA. This is shown in supporting Figure S4. The sequence analysis had shown a five-fold down-regulation of miR-205 in Thymus tissue as compared to EBV+-NK/T-cell lymphoma while the non-infected T-cell lymphoma showed no expression of this miRNA (Table 1).

### Identification of the Pro-inflammatory Cytokine IL1A as a Target of the Deregulated miR-142-3p

**Figure 3:**
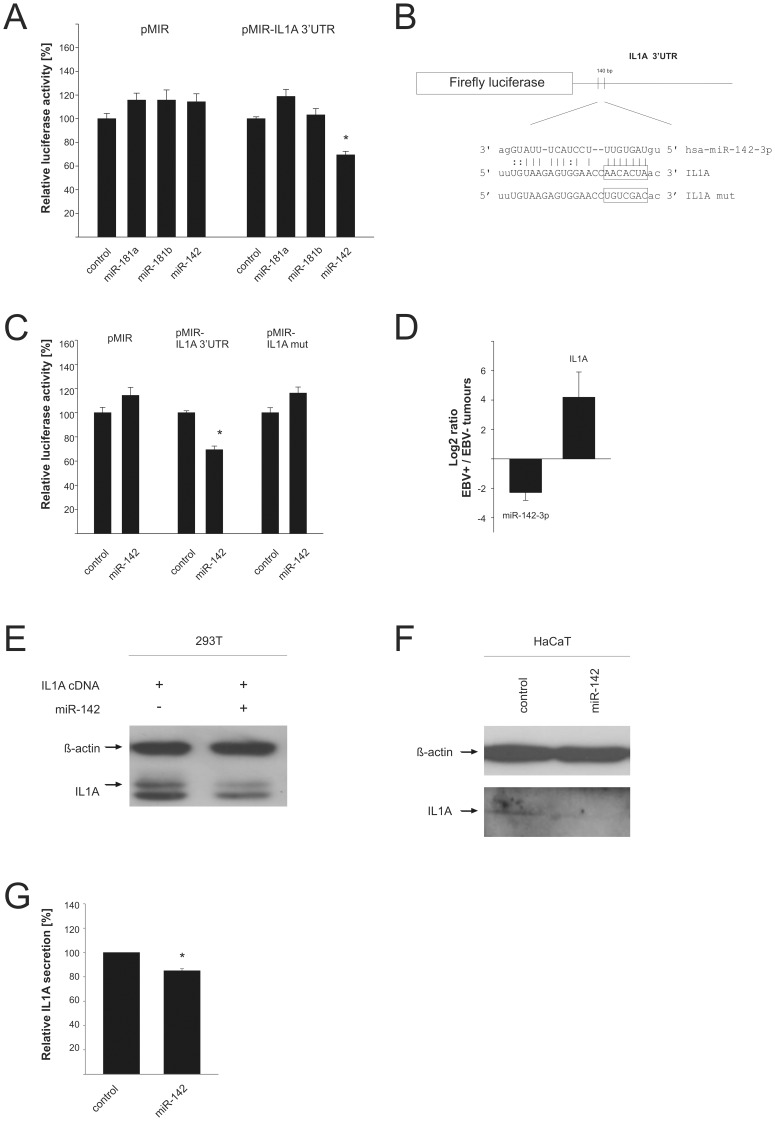
Regulation of IL1A by miR-142. A: A firefly luciferase reporter containing the 3′UTR of IL1A was cotransfected with different miRNAs predicted to target this 3′UTR or a control vector. The diagram represents five independent experiments with standard error carried out in duplicates. The luciferase activity of control transfected cells was set to 100%. B: Schematic overview about the predicted binding site of miR-142-3p in the 3′UTR of IL1A. The sequence in the 3′UTR mutated by site-directed-mutagenesis is indicated. C: Influence of miR-142 on the luciferase activity of the empty vector, the IL1A-3′UTR containing reporter as well as the mutated 3′UTR. The graph shows five independent experiments which were done in duplicates. D: Relative expression change of miR-142-3p and IL1A between EBV-associated NK/T-cell and EBV-negative T-cell lymphomas analysed by a quantitative Real-Time PCR presented as log2. Relative quantification was measured by analysing GAPDH and 5.8sRNA and calculated by the 2^−ΔΔct^ –method. The pool of miRNAs used for the construction of the cDNA libraries (4 each, see Materials and Methods section) was used for this analysis. E: Influence of mR-142 on the expression level of a full length IL1A cDNA clone in 293T cells. Extracts of 293T cells transfected with the miRNA expression vector or vector control were analysed by Western blotting with specific IL1A and ß-actin antibodies. The signals for ß-actin served as a loading control. F: Effect of miR-142 on the endogenous IL1A protein expression. Lysates of transfected HaCaT cells were analysed in a Western blot with IL1A- and ß-actin - specific antibodies. The signals for ß-actin served as a loading control. G: Influence of miR-142 on the secretion of IL1A. The IL1A-levels of supernatants of HaCaT transfected with miR-142 or vector control cells were analysed by ELISA. The diagram represents the results from three independent experiments carried out in duplicate.

We then went on to identify potential targets for the deregulated cellular miRNAs. A previous study had determined the mRNA expression profile of EBV-infected nasal NK/T-cell lymphoma lines and found, among others, the IL1A, BCL6, CD44 and c-FOS genes to be induced and the interleukin 1 receptor 1 (IL1R1) gene to be repressed compared to normal lymphocytes [18]. By applying a combination of various prediction algorithms, we identified the 3′-UTR of c-FOS as a potential target for miR-181a and miR-181b (down-regulated the in EBV-associated lymphomas), CD44 as a potential target of miR-20a and miR-106a, and the IL1R1 as a target of miR-205 and -125a. When we tested the 3′-UTRs of these genes in a luciferase reporter assay with the respective miRNAs, we did not observe an effect of any of these miRNAs on the reporter constructs (data not shown). Expression of the cloned miRNAs from the vector pSG5 is shown in supporting Figure S2. We further identified the 3′-UTR of the IL1A gene as a potential target for miR-142-3p, miR-181a and miR-181b which are repressed in EBV-associated lymphomas. The luciferase reporter analysis showed that miR-142-3p but not the other two miRNAs had a significant effect on the reporter. As shown in Figure 3A, we determined a down-regulation of the reporter construct by 31% (p = 4.3×10^–8^). The seed sequence of potential binding site for miR-142-3p on the IL1A 3′-UTR (boxed in Figure 3B) was then changed by site-directed mutagenesis. The mutated reporter lost its responsiveness towards miR-142-3p demonstrating that the IL1A 3′-UTR is a bona fide target of this miRNA (Figure 3C). We determined the relative levels of miR-142-3p and IL1A in the EBV-positive vs. -negative lymphomas and found an inverse relationship pointing at an effect of this miRNA vis-à-vis the IL1A 3′-UTR (Figure 3D). When we tried to express miR-142-3p in NK- or T-cell lines that were at our disposal by electroporation employing various systems, we got only a small percentage of transfected cells or a large number of apoptotic cells. That precluded a direct analysis by Western blotting or FACS analysis. We therefore transfected 293T cells with a full length cDNA clone which also comprised the 3′-UTR; as can be seen in Figure 3E, the band corresponding to IL1A was reduced to about 70% when miR-142-3p was co-expressed. We then analysed the HaCaT cell line which is known to express IL1A; transient expression of miR-142-3p also resulted in an approx. 30% reduction of the endogenous IL1A (Figure 3F). We analysed the effect of miR-142-3p on the amount of secreted IL1A in HaCaT cells. As shown in Figure 3G, the amount of IL1A in the supernatant of the miR-142-3p expressing cells was reduced by about 15% which is statistically significant (p = 0.0007).

### The Differentially Expressed miR-205 Targets the Oncogene BCL6

**Figure 4:**
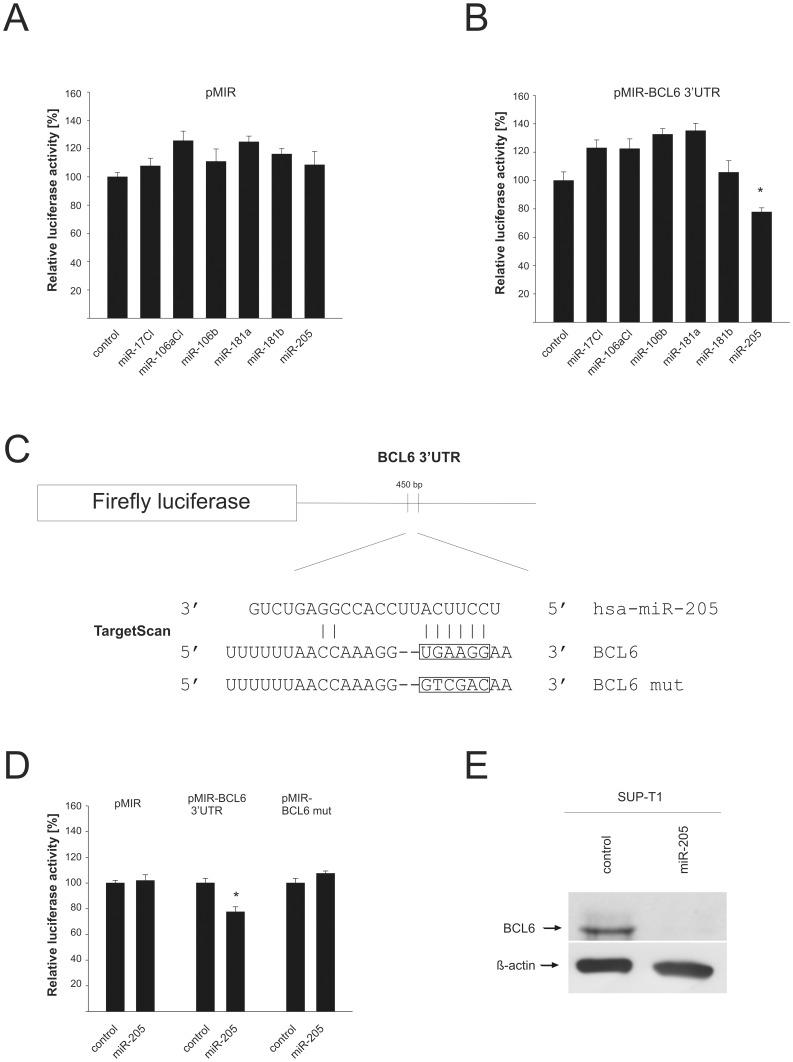
BCL6 is a target of miR-205. A: Effect of the analysed miRNAs on the empty vector. Cells co-transfected with the miRNA expression vectors, vector control and the empty reporter vector ware tested in luciferase assays. The diagram represents five independent experiments carried out in duplicate with the standard error indicated. The Renilla luciferase activity was used for normalization and firefly luciferase activity of control transfected cells was set to 100%. B: Regulation of the luciferase activity of the reporter construct containing the BCL6 3′UTR by several miRNAs predicted to target the 3′UTR. The bars represent four different experiments done in duplicate. C: Schematic overview of the predicted binding site of miR-205 in the 3′UTR of BCL6. The sequence in the 3′UTR mutated by site-directed-mutagenesis is indicated. D: Influence or miR-205 on the luciferase activity of the empty vector, the *wt*-3′UTR and the mutated BCL6 3′UTR. The diagram shows the results of four independent experiments carried out in duplicate. E: Transfection of miR-205 reduces the BCL6 protein in SUP-T1 cells. SUP-T1 cells transfected with miR-205 or a vector control were analysed in a Western blot with BCL6- and ß-actin- specific antibodies. The signals for ß-actin served as a loading control.

The computational prediction of potential targets had indicated that the 3′-UTR of the BCL6 proto-oncogene might be a target for miR-17, -106a, -106b, -181a, -181b, and -205. We generated expression clones for these miRNAs, either as single expression clones or within a miRNA cluster and tested these with the cloned BCL6-3′-UTR. As shown in Figure 4, the miRNAs had no effect on the empty reporter vector (Figure 4A), and only co-expression of miR-205 (Figure 4B) resulted in a significant down-regulation of the BCL6-3′-UTR reporter by approx. 22% (p = 0.001). The seed sequence of potential binding site for miR-205 on the BCL6 3′-UTR (boxed Figure 4C) was then changed by site-directed mutagenesis and assayed again with miR-205. The mutated reporter lost its responsiveness demonstrating that the BCL6- 3′-UTR is a target of this miRNA (Figure 4D). To show that miR-205 indeed reduces the protein level of BCL6, the EBV-negative SUP-T1 T-cell line was transfected with miR-205 expression plasmid and the level of BCL6 protein was determined by Western blot. The densitometric analysis of the BCL6 signal indicated a reduction of BCL6 protein by 30%. A representative blot is shown in Figure 4E.

To further analyse the differential expression of BCL6 in nasal NK/T-cell lymphoma vs. primary tissue, we compared the expression levels of BCL6 in CD56+ cells derived from two healthy donors with the mRNA levels in the EBV-positive cell lines SNK6, SNT10 and NK-92. Here we found a slight increase for the BCL6 mRNA in SNK6 while the other two cell lines expressed less BCL6 mRNA. The data for these two donors are shown in supporting Figures S5 and S6. The results are compatible with a down-regulation of BCL6 due to an up-regulation of the miR-205 which targets this mRNA. In summary, we identified two targets of two deregulated miRNAs in the NK/T-cell lymphoma.

## Discussion

To the best of our knowledge, this is the first miRNA profile of EBV-positive NK/T-cell lymphoma, T-cell lymphoma and normal thymus tissue established by deep sequencing of small RNA libraries as NK/T-cell lymphomas are virtually always EBV-positive [1]. Therefore, we used EBV-negative T-cell lymphoma for comparison. Two previous publications described a miRNA analysis of thymus tissue [19], [20]. The paper by Liang et al. (2007) used a PCR-based method to evaluate miRNA levels in various normal human tissues including thymus [19]. In their analysis, as in ours, miR-16 was one of the predominantly expressed miRNAs. Using miR-16 for normalization, we compared the values for the thymus tissues for those miRNAs that were deregulated in our lymphomas. We found that their data matched ours for some but not all miRNAs. The publication by Barad et al. (2004) used a micro array analysis of five normal human tissues including thymus and reported for thymus that miR-96, -182, -183 and -200a showed the strongest expression of all miRNAs tested [20]. These miRNAs were present in the thymus small RNA library analysed here but did not represent highly expressed miRNAs. We assume that the different methods used were responsible for the observed discrepancies.

When this analysis was designed, the nature of the non-neoplastic precursor cells of the NK/T-cell lymphoma was still a matter of debate. We therefore chose to analyse thymus tissue as a non-transformed control tissue [21] known to be involved in NK/T-cell development [22]. It is assumed today that most NK/T-cell lymphomas are derived from CD56^+^ NK cells or occasionally from cytotoxic T-cells [17]. Recently, Ng et al. (2011) analysed the miRNA levels of nasal NK/T-cell lymphoma and a panel of NK/T-cell lines in comparison to CD56+ precursor cells by an array-based technology. They observed a general down-regulation of cellular miRNAs with only a few up-regulated miRNAs of most miRNAs analysed [23]. Our data confirm these results in that we observe a general repression of miRNA expression as demonstrated by the reduced relative number of miRNA counts in the library of the EBV+-NK/T-cell lymphoma (Figure 1) in line with our previous analyses of such libraries obtained for EBV-positive nasopharyngeal carcinoma (NPC) and diffuse large B-cell lymphoma (DLBCL) [11], [24]. Other publications have also described a global repression of miRNAs in tumours and that this repression can enhance tumorigenesis [25], [26].

One of the miRNAs which showed a reduced expression in both EBV-positive NK/T-cell lymphoma as well as the EBV-negative T-cell lymphomas was miR-218. The repression of miR-218 was already described for gastric carcinoma and shown to function as an inhibitor of invasion and metastasis [27]. Furthermore, the reduction of miR-218 was associated with strong activation of NFkB which is also often seen in NK/T-cell lymphoma [28]. The paper by Ng et al (2011) did not report a change in expression for miR-218, though [23]. In the EBV-negative lymphomas, the members of the miR-200 family were not expressed or showed only a very low expression. For most of them, the expression was also reduced in EBV-positive lymphomas compared to thymus; only miR-200b and miR-141 were slightly above the two-fold reduction. The repression of these miRNAs coincides with publications showing that the down-regulation of miR-200 family contributes to metastasis of tumour cells by targeting ZEB1/ZEB2 and increasing E-cadherin [29].

MiR-424 and -128a+b were significantly down-regulated in both tumour entities as compared to thymus. The down-regulation of miR-424 was essentially confirmed by qRT-PCR in the NK/T-cell lines and tumor tissue (supporting Figure S4). We had previously detected also a down-regulation of miR-424 in prostate carcinoma [30]. MiR-424 was linked to breast cancer as it is part of an oestrogen-responsive gene network in breast carcinoma cell lines [31]. The down-regulation of miR-142-5p and miR-142-3p in the EBV-positive NK/T-cell lymphomas as compared to the EBV-negative lymphomas was also confirmed by the qRT-PCR analysis including a comparison between CD56+ primary cells and the NK/T-cell lines and the tumor tissues (supporting Figure S4). Ng et al. also found a strong down-regulation of this miRNA [23]. A computational search yielded the mRNA of the interleukin 1 alpha (IL1A) gene as a potential target for this miRNA. Concomitant with the decrease in miR142-3p, the mRNA levels for IL1A were up-regulated in EBV-positive lymphomas and we could indeed prove that the IL1A 3′-UTR contains a binding site for this miRNA. Accordingly, miR-142 negatively regulated a reporter construct featuring the IL1A-3′-UTR which lost its responsiveness upon binding site mutation. Lastly, we could demonstrate that miR-142, when ectopically expressed in a human cell line, reduced the protein levels of IL1A and also reduced the levels of secreted IL1A. The up-regulation of IL1A was previously shown in EBV-positive NK/T-cell lymphomas along with mRNAs of other genes thought to be involved in cell proliferation such as BCL6 or ERBB4 [18]. The up-regulation of IL1A in the EBV-positive as compared to the EBV-negative lymphomas might be explained by the ability of the pro-inflammatory cytokine to act as an auto- or paracrine growth factor and as an inhibitor of apoptosis (for review, see [32]). Besides, this cytokine is known to be induced yet in several tumours, e.g. in NPCs, and to have tumour promoting activity [33]. Except for the direct regulation of IL1A by miR-142-3p found in this study, the miRNA has also an indirect influence on IL1A. It was already reported that AC9 is regulated by miR-142-3p and that the inhibition of AC9 resulted in a reduced amount of cAMP [34]. As cAMP in turn is able to induce the amount of IL1A, miR-142-3p is able to influence IL1A indirectly by regulating AC9, too [35]. We show that IL1A is a target for miR-142-3p and that the down-regulation of this miRNA might confer a selective advantage to the EBV-infected cell by elevating IL1A levels.

As mentioned, the BCL6 mRNA was also up-regulated in NK/T-cell lymphomas [18]. It should be pointed out that other studies did not find a deregulation of either IL1A or BCL6 [23], [36]. A computational analysis indicated the BCL6-3′-UTR as a potential target amongst others for miR-205 which was found here to be down-regulated in the EBV-positive NK/T-cell lymphomas and the EBV-negative samples as compared to normal tissue. The co-expression of miR-205 with a reporter construct featuring the BCL6-3′-UTR showed that this 3′-UTR is a target for miR-205; mutation of the binding site led to non-responsiveness of the reporter. Furthermore, ectopic expression of miR-205 resulted in down-regulation of the BCL6-protein level. BCL6 was originally discovered in B-cell lymphomas due to chromosomal translocations or mutations affecting this gene [37]. More recently, BCL6 was also found to play a role in the development of T-cell lymphomas [38], [39]. The BCL6 oncogene is a transcriptional repressor; e.g. it blocks the expression of the tumour suppressor proteins PDCD2 and p53 [40], [41] as well as the cell cycle inhibitor p21^kip^ [42]. Our data are compatible with the role of miR-205 in repression of BCL6 in normal tissue as expression of miR-205 was higher in thymus than in lymphoma tissues; in addition, miR-205 was virtually undetectable in EBV-negative lymphomas. However, we found only a slight induction of BCL6 in the NK/T-cell line SNK6, and a down-regulation in SNT10 and NK-92. This issue needs to be clarified in further studies.

We also found an induction of several miRNAs that could mostly be verified in primary tumours (see below). Of the cellular microRNAs described by Ng et al. that were induced in the tumors/cell lines [23], we also observed an up-regulation of miR-155 and miR-378 in the tumors vs. normal tissue. The sequence analysis demonstrated that miR-449a+b were the only miRNAs that were exclusively present in the EBV-positive samples. This miRNA was induced in endometrioid adenocarcinomas and adrenal hyperplasia [43], [44], but was also implicated in inhibition of cell-cycle progression and induction of apoptosis [45]. The up-regulation of miR-145 in EBV-associated NK/T-cell lymphoma was rather surprising as this miRNA is considered to have tumour suppressive functions [46]. However, a re-analysis by qRT-PCR showed that miR-145 was down-regulated both in the NK/T-cell lines and the tumor tissue tested (supporting Figures S4 and S5) in line with a tumor-suppressive function for miR-145 (see, for instance [47]).

Of the viral miRNAs, all but those derived from the BHRF1 cluster were detectable. MiRNAs derived from the BHRF1 cluster of EBV were reported to repress the chemokine CXCL-11 in DLBCL of immune-compromised patients with an EBV latency type III [14]. In contrast, the lymphomas in our study were derived from immune-competent, HIV negative patients. In accordance with the patients’ immune status, the EBV-positive nasal NK/T-cell lymphomas analysed in the present study did not express LMP or EBNA2 corresponding to an EBV latency type I. In addition, miRNAs derived from the BHRF1-cluster were not found as the type I latency does not appear to support expression of these transcripts. This observation matches the situation described for NPC [11], [48], gastric carcinoma [15] and peripheral T-cell lymphoma [49]. The absence of these miRNAs was also reported for an EBV-positive type I Burkitt’s lymphoma cell line [6], but so far, no data were available for primary nasal NK/T-cell lymphoma. Of note, the previously described EBV-miRNAs miR-BART-21 and -22 were both present in the lymphoma samples pointing at a role for these miRNAs for EBV function. We recently published that the viral miRNAs constituted 5–19% of all miRNAs in EBV-infected NPC with BART4 showing the strongest expression [11]; in NK/T-cell lymphoma, the viral miRNAs represented 2.3% of the total miRNA reads with BART-7, -5, -11-5p, 1-5p and -19-3p accounting for 50% of the viral miRNAs which amount to about 1% of the total miRNA.

A distinct advantage of the procedure employed here is that there is a potential to identify novel miRNAs as compared to a micro-array or a PCR-based analysis. Indeed, we were able to identify 10 novel miRNAs from known precursors as well as three so far unknown miRNA precursors. While writing this article, miR-pot.42 was published as miR-3157 at miRBase. The analysis of the potential precursors for the two remaining miRNAs using the “mfold” program (http://bibiserv.techfak.uni-bielefeld.de/rnahybrid) showed an alignment into the expected hairpin structures. In addition, the two predicted miRNAs were conserved between various vertebrate species, for instance in chimpanzee and gorilla. According to previously set standards [50], the sequencing, the alignment in hairpin structures and the inter-species conservation establish the three sequences as novel miRNAs. We only obtained 2 reads for each of the three potential miRNAs which is a sign of low abundance and explains the failure to get a signal in the northern blot analysis of NK/T-cell lymphoma lines.

In summary, we describe for the first time a comprehensive miRNA analysis of EBV-positive NK/T-cell lymphomas by deep sequencing and demonstrate the relevance of deregulated miRNAs for the post-transcriptional gene regulation in these lymphomas. Likewise, we present an analysis of the miRNA expression in thymus tissue as well as in EBV-negative T-cell lymphomas. These data will help to acquire a better understanding of the processes underlying lymphoma development and may help to design rational strategies for treatment, i.e. via antisense methodologies or by reintroduction of miRNAs into tumour cells.

## Materials and Methods

### Patients and Immunohistochemistry

For the lymphoma samples used in this study diagnosis was performed on paraffin embedded tissue according to the WHO classification 2008. Corresponding snap frozen lymphoma tissue samples where collected at the Institute of Surgical Pathology, University Hospital of Zurich. Five immunocompetent patients with EBV-negative T-cell and two with EBV-associated nasal NK/T-cell lymphomas were included in the study. Care was taken to insure that the tumor content was at least 60–70% in the samples used to generate the libraries. The use of patient’s material and the study was approved by the ethical committee of the Canton of Zürich, Zürich, Switzerland (Reg.-Nr. StV2-2007). Written consent was obtained from all patients for the use of materials.

Immunohistochemical stainings and EBER in situ Hybridization were performed on an automated stainer (Ventana, Medical Systems, Tucson, AZ, USA), using the antibodies LMP (Clone CS1-4; Cell Marque, Rocklin, USA/California), EBNA2 (Clone PE2; Dako, Glostrup, Denmark) and Zebra (Clone BZ.1; Dako, Glostrup, Denmark). EBV encoded small RNA (EBER) was hybridized to the inform EBER probe cocktail and stained with ISH iVIEW nitro blue Tetrazolium (Ventana Medical Systems, Tucson AZ, USA).

### Small RNA Cloning

The small RNA fraction from five EBV-negative T-cell lymphomas, two EBV-associated NK/T-cell lymphomas and four thymus tissues, respectively, were pooled and three small RNA libraries were generated. Small RNA cloning, sequencing and analysis was carried out exactly as described previously [11].

### Sequence Annotation

The sequence data was analysed with the help of Excel macros. The analysis was done semi-manually because poly-a-signals disturbed the 454 sequencing resulting in “A”-insertions. For sequence analysis first the poly-a-tail as well as the adapter- and barcode sequences had to be removed. The resulting reads were compared to a miRNA-database comprised of all mature known mature miRNA-sequences from miRBase v12.0. Because we chose less stringency there were a lot of false negatives. Therefore the resulting sequences were identified afterwards by using the search function of the database miRBase. The leftover non-miRNA sequences were then tried to be assigned to a ncRNA-database consisting of rRNA, tRNA, snoRNA, snRNA etc. retrieved from fRNAdb:Blast (http://www.ncrna.org/frnadb/blast, http://gene.fudan.sh.cn/), sno/scaRNAbase (snoRNAbase.nsf). The sequences which could still not be identified by this comparison were aligned against human or EBV encoded transcripts (accession umber AJ507799) by blasting (NCBI Nucleotide Blast). The miRNA expression of the different tissues was compared by relative miRNA expression. The relative expression is the absolute read number of a single miRNA normalized to the absolute number of all expressed miRNAs in a small RNA library.

### Identification of New miRNAs

With the help of the search function of miRBase v12.0 new mature miRNAs from already known precursors could be identified. The genomic regions of potentially new miRNAs with 150 nt flanks were retrieved from UCSC Genome Browser (http://genome.ucsc.edu/) to fold the sequence to secondary structures by RNAhybrid (http://bibiserv.techfak.uni-bielefeld.de/rnahybrid). Only sequences that folded into hairpins and where the hairpins were conserved in another species were considered as potentially new miRNA sequences.

### Cell Culture

SUP-T1 [51], Jijoye [52] and BL41-B95.8 [53] cells were cultured in RPMI 1640 supplemented with 10% fetal calf serum (FCS) and 0.1% antibiotics. HANK-1 [54] was grown in MEM/RPMI1640 supplemented with 5% human plasma, 1xInsulin-Transferrin-Selen-X-Supplement (Invitrogen), 0.1% antibiotics and 100 U/ml IL-2. NK-YS [55] were cultured in IMDM with 10% FCS, 1% Kanamycin and 100 U/ml IL-2. NK-92 cells [56] were grown in X-Vivo Medium supplemented with 5% human plasma, 0.5% Penicillin/Streptomycin and 500 U/ml IL-2. The cultivation of 293T and HaCaT cells was carried out in DMEM with 10% FCS and 0.1% antibiotics [30]. HaCaT cells [57] were obtained from S. Smola, Institut of Virology, University Hosptial of the Saarland, Homburg, Germany, 293T cells were cultured as described previously [30].

### Isolation of CD56+ Cells

PBMCs were isolated from buffy coats (obtained from the Blutspendezentrale Saar-Pfalz GmbH, Germany) by ficoll separation. CD56^+^ cells were isolated from PBMCs by positive selection using human CD56 MicroBeads on a MACS separator (Miltenyi Biotec, Bergisch Gladbach. Germany). Total RNA was isolated from CD56^+^ cells using Trifast (Peqlab, Erlangen, Germany).

### Northern Blot

Total RNA was isolated from Jijoye, BL41-B95.8, Hank-1, NK-YS, NK-92 and SUP-T1 cells using Trifast (Peqlab, Erlangen, Germany) according to the manufacturer’s protocol, separated on a 12% urea-polyacrylamid gel and transferred to nylon membrane Hybond N (Amersham, München, Germany) for 30 minutes at 15V. The RNA was chemically cross-linked for 2 h at 55°C [58]. After hybridization with radioactive labeled antisense RNA probes overnight, the blots were washed twice for 15 minutes with 5×SSC, 1% SDS and twice for 15 minutes with 1×SSC, 1% SDS. The RNA Probes were generated with miRVana Probe construction kit (Ambion, Austin, USA) according to the manufacturer’s instructions.

### Reverse Transcription and Quantitative Real-time PCR

DNaseI treated RNA was first polyadenylated with the Poly-A-tailing kit from Ambion (Austin, USA). After an isolation step with Trifast (Peqlab, Erlangen, Germany) the RNA was reversed transcribed using a Poly-(T)-adapter primer (5′-GCGAGCACAGAATTAATACGACTCACTATAGG(T)12VN*-3′) and the SuperScript™ III First strand synthesis system (Invitrogen, Carlsbad, USA). Semi-quantitative RT-PCR was carried out with the Light-Cycler 1.5 System. MiRNAs were amplified using the LightCycler® FastStart DNAMasterPLUS SYBR Green I Kit (Roche Applied Science, Mannheim, Germany) and a reverse primer (5′-GCGAGCACAGAATTAATACGAC-3′) as well as miRNA specific forward primers (miR-200c: 5′-TAATACTGCCGGGTAATGATGGA-3′, miR-205: 5′-TCCTTCATT CCACCGGAGTCTG-3′, miR-145: 5′-GTCCAGTTTTCCCAGGAATCCCTAA-3′, miR-148a: 5′-TCAGTGCACTACAGAACTTTGT-3′, miR-142-3p: 5′-TGTAGTGTTTCCT ACTTTATGGA-3′, miR-181b: 5′-AACATTCATTGCTGTCGGTGGGT-3′, miR-424: 5′-CAGCAGCAATTCATGTTTTGAA-3′, miR-20b: 5′-CAAAGTGCTCATAGTGCAG GTAGAA-3′). For relative quantification 5.8sRNA was used (5.8sRNA: 5′-CTACGCCTGTCTGAGCGTCGCTT-3′). The amplification of IL1A (for: 5′-GGTTGA GTTTAAGCCAATCCA-3′, rev: 5′-TGCTGACCTAGGCTTGATGA-3′) and GAPDH (5′-GGTTGAGTTTAAGCCAATCCA-3′, 5′-GGTTGAGTTTAAGCCAATCCA -3′) was carried out with the universal probe library (Roche Applied Science, Mannheim, Germany). Relative expressions were calculated according to 2^(−ΔΔc^ *_T_* ^)^- method [59].

### Oligonucleotides and Plasmids

To test 3′UTRs in luciferase-assays, the complete 3′UTRs were cloned in the modified vector pMIR-RNL described previously [11]. The following primers were used for PCR-amplification of the 3′UTRs: 5′IL1A 5′-GACTAGTCTACTGGGTGTGCTTGGCA-3′, 3′IL1A 5′-CGAGCTCCATTATGGTCTGAT CAC-3′, 3′BCL6 5′- CGGCTAGCGAATTCAGCCAAACCCTGTCTCCGG-3′, 5′BCL6 5′- GCTCTAGATTCCGTCACAAAAGCCAGCT-3′. The mutation of the miRNA binding site in the 3′UTR was done with a site-directed mutagenesis and the following primers: 5′IL1A 5′-TAAGAGTGGAACCTGTCGACACATATAATGTTGTT-3′, 3′IL1A 5′-ACAACATTATATGTGTC GACAGGTTCCACTCTTACA-3′, 5′BCL6 5′-TTTAACCAAAGGGTCGACAATATATGGCA GAGTTG-3′, 3′BCL6 5′-CAACTCTGCCATATATTGTCGACCCTTTGGTTAAA-3′.

For the expression of miR-205, the primers 5′miR205-Eco 5′GGAATTCCGGGTAGGAGTATTCAGGTCC-3′ and 3′miR205_Bam 5′-CGGGATCCTCCCTCTGAAGAAGCACGCA-3′ were used to amplify the genomic luus (416bp) that were then inserted in the pSG5 expression vector [24]. Likewise, miR-142 was amplified using the primers 5′miR142_Eco 5′-GGAATTCGGGATCTTAGGAAGCCACA-3′ and 3′miR142_Bam 5′-CGGGATCCATGGAGGGCCTTTCAGGCAT-3′. For the expression of miRNAs miR-125a, -181a, -181b, -106a, -106b, and 17, the following primer pairs were used: 5′miR-125a_EcoCGGAATTCTGGCTCTCAGAATGTCTC-3′, 3′miR-125a_Bam 5′-CGGGATCCGCCATCGTGTGGGTCTCAA-3′; 5′miR-181a-Bam 5′-GCGGATCCTGTGATGTGGAGGTTTGCC; 3′miR-181a-Bgl 5′-GCAGATCTAGTGAGCTTGTCCACACAG-3′; 5′miR-181b-Bam 5′-GCGGATCCCAACGCTGTCGGTGAGTT-3′, 3′miR-181b-Bgl 5′-CAGATCTGCATGGGTGCTGAGGTCCT; 5′miR-17Eco 5′-GGGAATTCCGTGTCTAAATGGACCTC-3′, 3′miR-17-Bam 5′-GGGATCCACAGCATTGCAACCGATCCCAA-3′; 5′miR-106a Eco 5′-GCGAATTCGCTTAGACTCTGTAAGCC-3′, 3′miR-106a-Bam 5′-GGGATCCTACGCTGAAATGCAAACCTGC-3′; 5′miR-106b Eco 5′-GCGAATTCTGGTAAGTGCCCAAATTGCTGG-3′; 3′miR-106b Bam 5′-GGATCCAGCACAGGATCTAGGACACATG-3′; 5′potmiR-27 Eco 5′-GCGAATTCTGGAGCTCATGAAGAGACCAAG-3′, 3′potmiR-27 Bgl 5′-GGAAGATCTAGGACAGTCTGTGTCCTCAG-3′; 5′potmiR-34 Eco 5′-GCGAATTCTGCTGTGTCAGAAAGGCTTCAC-3′, 3′potmiR-34 5′-GCGGATCCTGGGCATTCTTTCATCCCATC-3′; 5′ potmiR-42 Eco 5′-GCGAATTCGTCTGTATTCTCTTCTGGC-3′; 3′potmiR-42 Bam 5′-GGATCCCTGCTTTGAGAGTTCCTGAGT-3′. The expression from the corresponding pSG5 plasmids was analzed by Northern blotting (shown in supplementary Figure S2).

### Luciferase Assays

For luciferase assays, 200 ng of reporter and 800 ng of miRNA expression plasmid were transfected 24 h after seeding 293T cells in 24-well plates using Nanofectin (PAA, Cölbe, Germany) following the manufacturer’s protocol. Luciferase activity was measured 48 h after transfection using the dual luciferase reporter assay (Promega, Mannheim, Germany). The pMIR dual-luciferase reporter vector was described previously [24].

### Western Blotting

HaCaT cells were transfected with 2 µg plasmid 24 h after seeding 3×10^5^ cells in 6-well plates. The transfection was done with Metafectene with a ratio 1∶6 (DNA/Metafectene). 2×10^6^ SUP-T1 cells were transfected with a nucleofector^®^ (Lonza, Köln, Germany) by using 2 µg DNA, the nucleofector solution V and the program O-017. 293T cells were seeded in 6-well plates and transfected 24 h later with 1,5 µg IL1A-full length cDNA and 1,5 µg pSG5/miRNA by using Nanofectin (PAA, Cölbe, Germany). 48 h after transfection cells were lysed with sample buffer. Extracted proteins (30 µg) were separated on a 12.5% polyacrylamide gel and transferred to a Protran™ nitrocellulose membrane (Roth, Karlsruhe, Germany). After blocking the membrane with 5% milk in PBS, the membranes were incubated with the following primary antibodies: anti-human-IL1A Clone 4414 (R&D Systems, Minneapolis, USA), anti-human BCL6 clone N3 (Santa Cruz biotechnology, Santa Cruz, USA), and anti-human ß-actin (Sigma, München, Germany). The secondary anti-mouse antibodies used in this study were coupled to horseradish peroxidase.

### ELISA

The supernatant of HaCaT cells transfected with 2 µg plasmid 24 h after seeding 3×10^5^ cells in 6-well plates was collected 48 h after transfection. The supernatants were stored at −80°C after centrifugation for 10 min at 3000 rpm. The secreted IL1A was detected by the human IL-1alpha/ILF1 Duo Kit (R&D Systems, Minneapolis, USA) according to the manufacturer’s protocol. The measurement was done with a multiplate reader Victor X™ (Perkin Elmer, Rodgau, Germany) at wavelengths of 450 nm and 550 nm. The data analysis was carried out by the software WorkOut 2.5 with the 4-Parameter method.

## Supporting Information

Figure S1 **Analysis of miRNA expression by Northern Blot. A:** Analysis of BHRF1-miRNAs in the EBV-positive NK/T-cell lymphoma cell lines by Northern Blot. Total RNA was separated in a polyacrylamid gel and transferred to a nylon membrane. After chemical cross linking, the mature miRNAs were detected with radioactively labelled RNA probes. RNA from EBV-infected B-cell lines served as a positive control whereas RNA from EBV-negative NK-92 and SUP-T1 served as negative control. In the EBV-associated NK/T-cell lymphoma cell lines NK-YS and HANK-1, no expression of the BHRF1-miRNAs could be observed. **B:** Northern Blot analysis of the potential new miRNAs in NK/T-cell lines. Total RNA from 293T cells transfected with a miRNA expression construct served as a positive control. The band representing the tRNA served as loading control.
(TIF)

Figure S2 **Expression control of generated miRNA expression constructs.** Expression of novel miRNAs was analyzed in 293T cells transfected with the indicated miRNA expression constructs. The tRNA served as loading control.
(JPG)

Figure S3 **Comparison of miRNA levels in cell lines/tumors vs. Thymus.** The miRNA expression levels in one additional primary case (“NKTL St. Gallen”), three additional cases of NKTL were formalin–fixed paraffin-embedded (FFPE) material was available, and the NKTL cell lines SNK6 and SNK10 were compared to thymus tissue. The value obtained for Thymus tissue was set to 1 and the relative change is indicated. The graphs represent the mean values of three experiments carried out in duplicate.
(JPG)

Figure S4 **Comparison of miRNA levels in cell lines/tumors vs. CD56+ primary cells.** The same tissues and cell lines as described in Figure S3 were used and compared to the value obtained for pooled CD56+ cells isolated from 5 healthy donors. The graphs represent the mean values of three experiments carried out in duplicate.
(JPG)

Figure S5 **Comparison of BCL6 mRNa levels in CD56+ vs NKTL tumor cell lines.** The mRNA levels of the BCL6 gene in non-transformed CD56+ cells isolated from healthy donor 1 were compared with the NKTL lines SNK6, SNK10, and NK-92. The graphs represent the mean values of three experiments carried out in duplicate.
(JPG)

Figure S6 **Comparison of BCL6 mRNa levels in CD56+ vs NKTL tumor cell lines.** The mRNA levels of the BCL6 gene in non-transformed CD56+ cells isolated from healthy donor 2 were compared with the NKTL lines SNK6, SNK10, and NK-92. The graphs represent the mean values of three experiments carried out in duplicate.
(JPG)

Table S1 **Absolute and relative EBV-miRNA expression in NK/T- cell lymphoma by sequencing.**
(DOC)

Table S2 **Differentially expressed miRNAs in EBV-negative T-cell lymphoma compared to Thymus.**
(DOC)

Table S3 **Differentially expressed miRNAs in EBV-positive T-cell lymphoma compared to Thymus.**
(DOC)

Table S4 **Absolute and relative miRNA expression in the small RNA libraries analysed by sequencing.**
(DOC)

Table S5 **New miRNAs from unknown precursors identified from EBV-negative T-cell lymphoma.**
(DOCX)
