# Supplementary figures and images for: MicroRNA Profiling of Epstein-Barr Virus-Associated NK/T-Cell Lymphomas by Deep Sequencing
Source: PLoS One. 2012 Aug 3;7(8):e42193. doi: 10.1371/journal.pone.0042193 (PMC3411711; doi:10.1371/journal.pone.0042193)

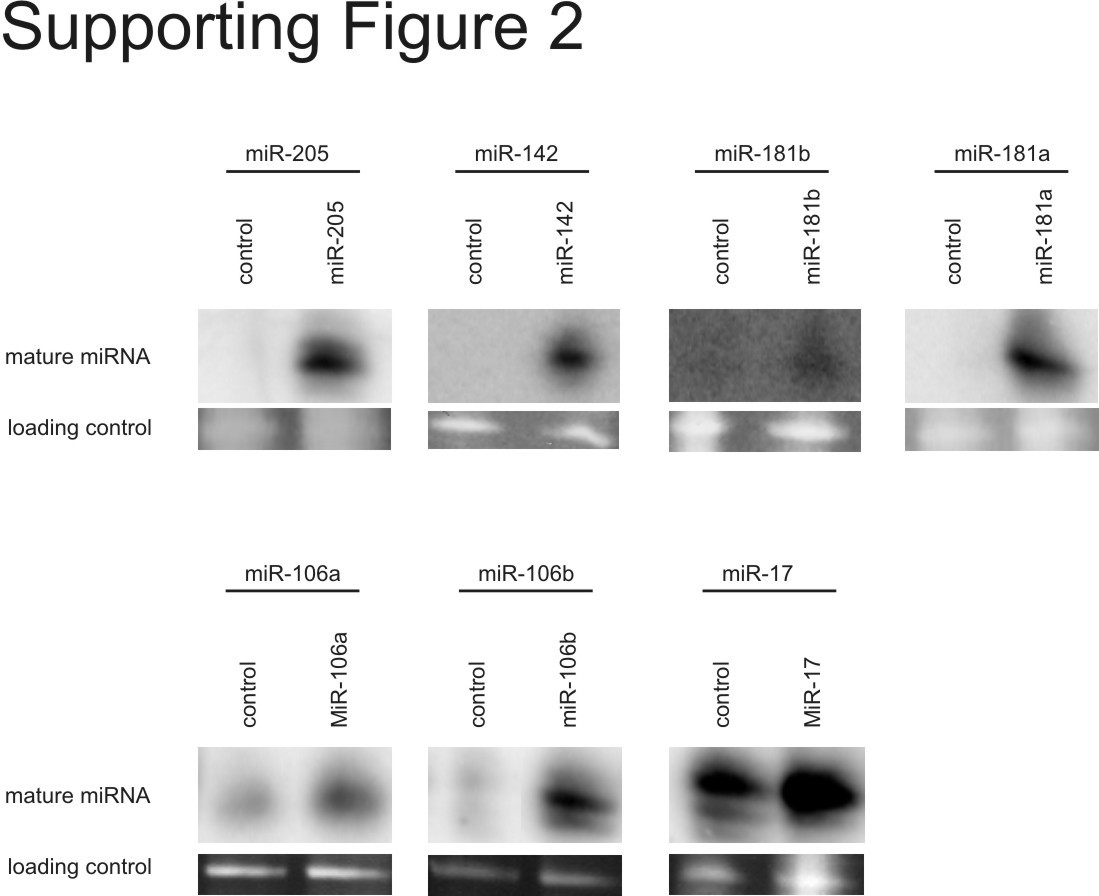

Supplement: Figure S2 — Expression control of generated miRNA expression constructs. Expression of novel miRNAs was analyzed in 293T cells transfected with the indicated miRNA expression constructs. The tRNA served as loading control. (JPG) [file pone.0042193.s002.jpg]

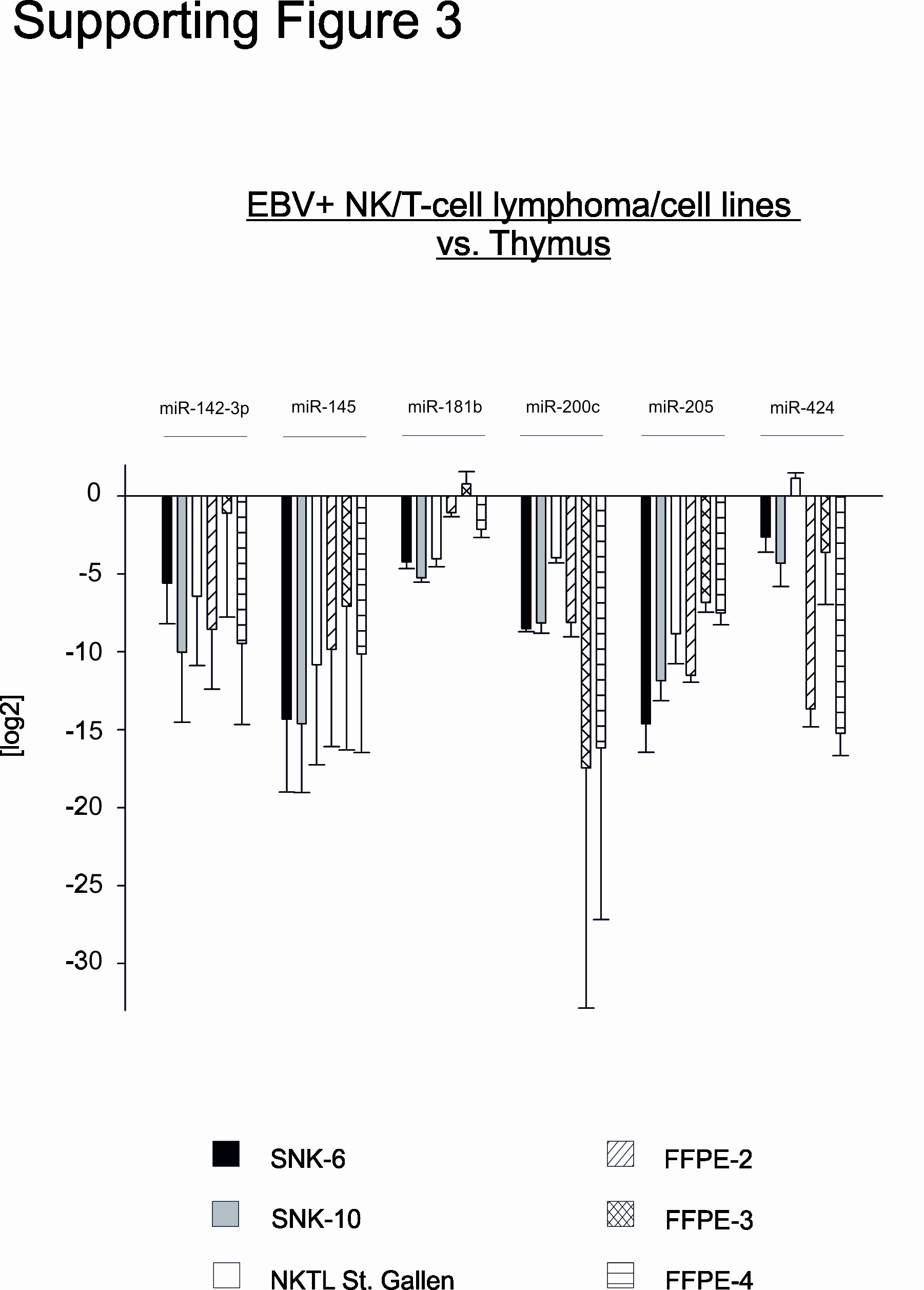

Supplement: Figure S3 — Comparison of miRNA levels in cell lines/tumors vs. Thymus. The miRNA expression levels in one additional primary case (“NKTL St. Gallen”), three additional cases of NKTL were formalin–fixed paraffin-embedded (FFPE) material was available, and the NKTL cell lines SNK6 and SNK10 were compared to thymus tissue. The value obtained for Thymus tissue was set to 1 and the relative change is indicated. The graphs represent the mean values of three experiments carried out in duplicate. (JPG) [file pone.0042193.s003.jpg]

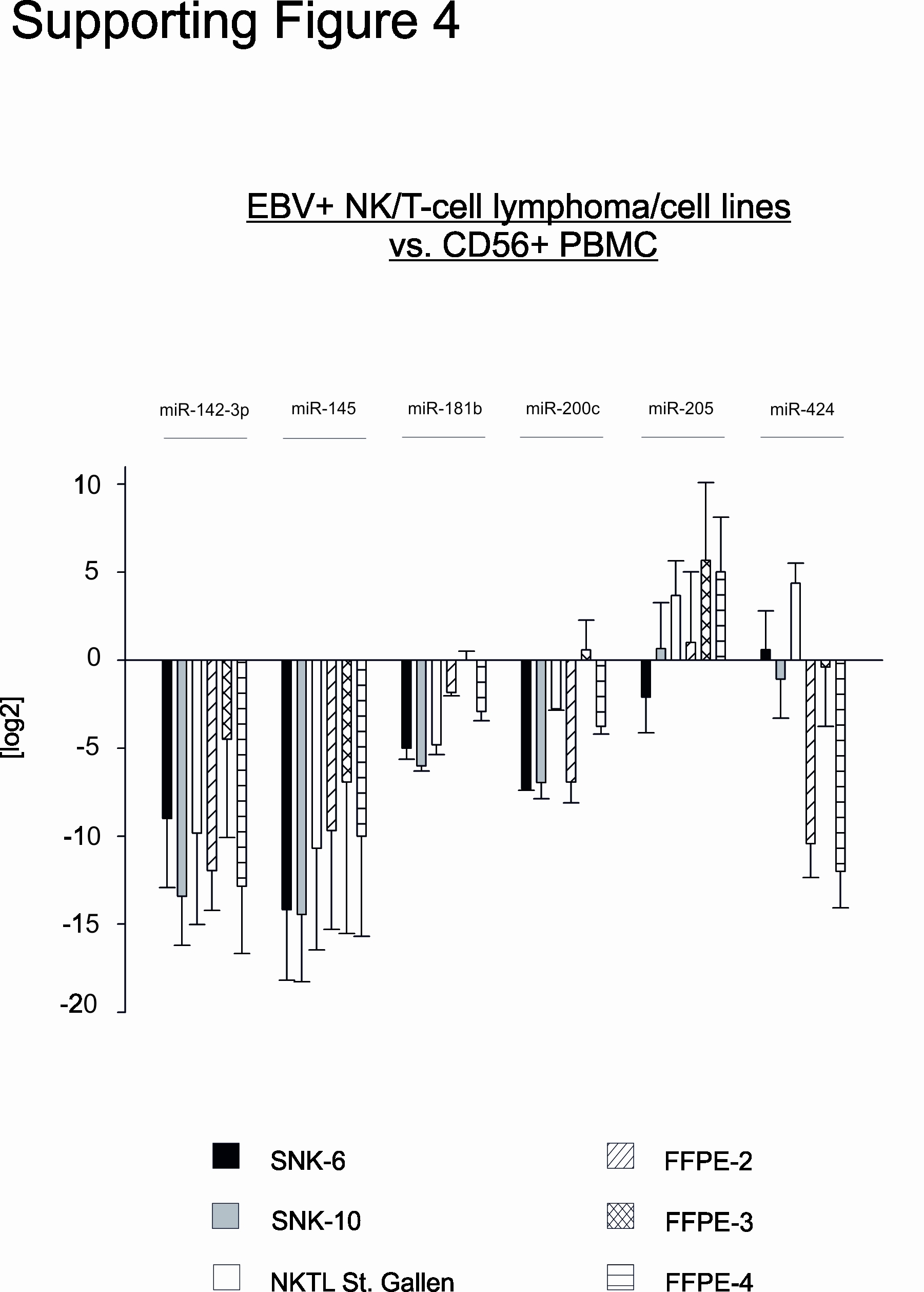

Supplement: Figure S4 — Comparison of miRNA levels in cell lines/tumors vs. CD56+ primary cells. The same tissues and cell lines as described in Figure S3 were used and compared to the value obtained for pooled CD56+ cells isolated from 5 healthy donors. The graphs represent the mean values of three experiments carried out in duplicate. (JPG) [file pone.0042193.s004.jpg]

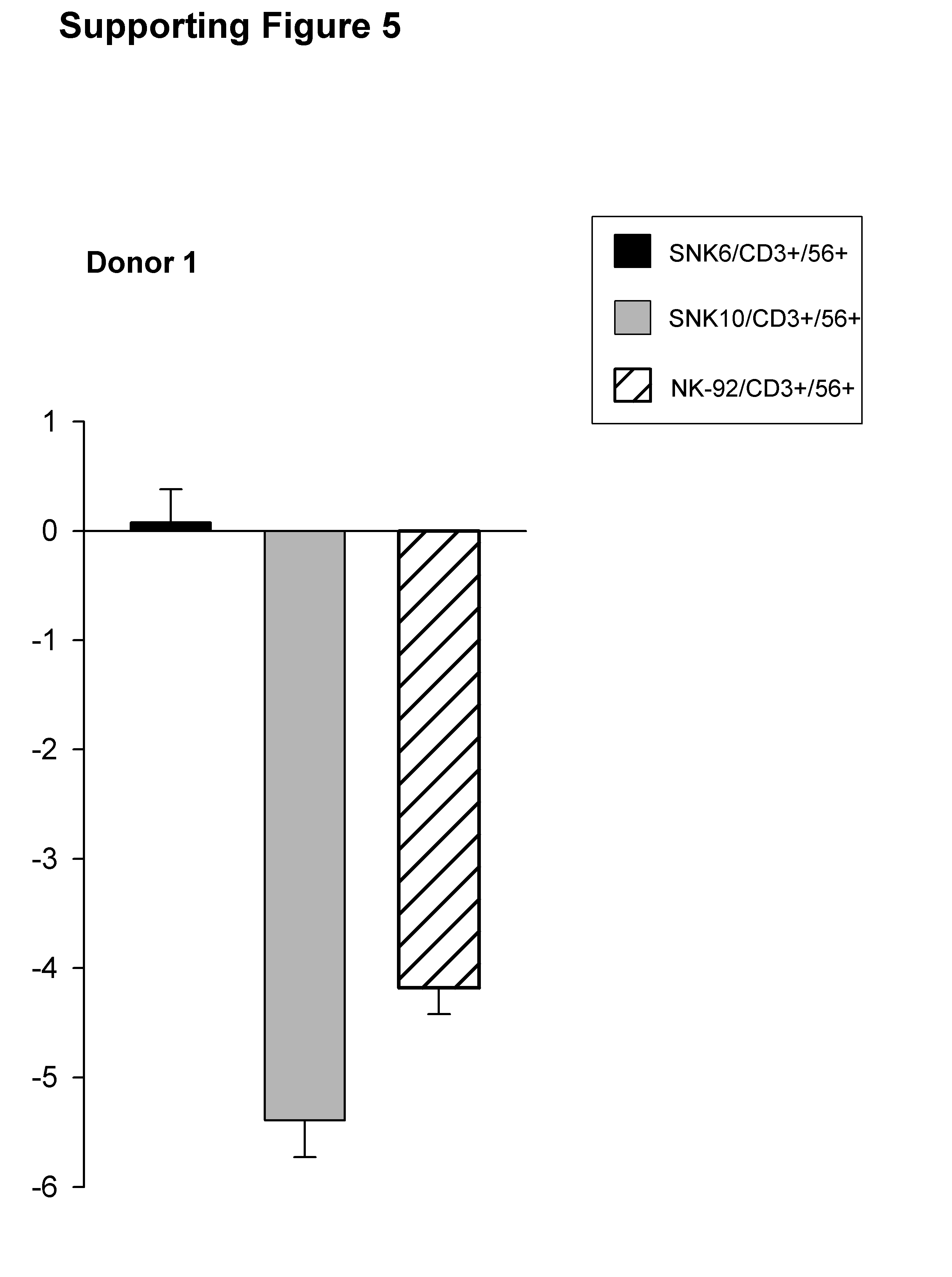

Supplement: Figure S5 — Comparison of BCL6 mRNa levels in CD56+ vs NKTL tumor cell lines. The mRNA levels of the BCL6 gene in non-transformed CD56+ cells isolated from healthy donor 1 were compared with the NKTL lines SNK6, SNK10, and NK-92. The graphs represent the mean values of three experiments carried out in duplicate. (JPG) [file pone.0042193.s005.jpg]

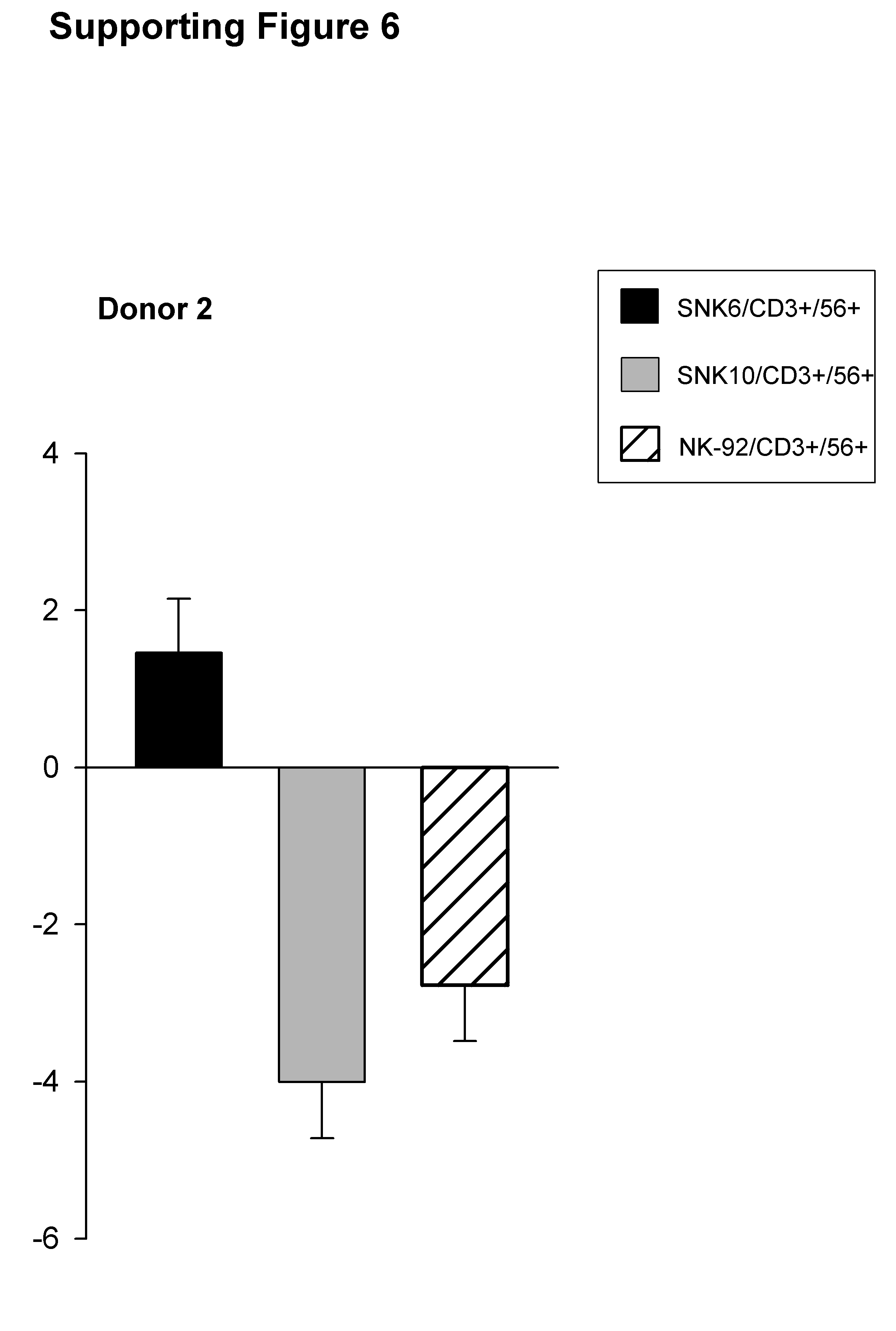

Supplement: Figure S6 — Comparison of BCL6 mRNa levels in CD56+ vs NKTL tumor cell lines. The mRNA levels of the BCL6 gene in non-transformed CD56+ cells isolated from healthy donor 2 were compared with the NKTL lines SNK6, SNK10, and NK-92. The graphs represent the mean values of three experiments carried out in duplicate. (JPG) [file pone.0042193.s006.jpg]
