# Supplementary material for: MicroRNA Profiling of Epstein-Barr Virus-Associated NK/T-Cell Lymphomas by Deep Sequencing
Source: PLoS One. 2012 Aug 3;7(8):e42193. doi: 10.1371/journal.pone.0042193 (PMC3411711; doi:10.1371/journal.pone.0042193)
Supplement: Table S1 — Absolute and relative EBV-miRNA expression in NK/T- cell lymphoma by sequencing. (DOC) [file pone.0042193.s007.doc]

**Supporting Table S1**

| **miRNA** | **total reads of microRNA** | **rel.miRNA expression [%]** | **rel.EBV-miRNA expression [%]** |
| --- | --- | --- | --- |
|  |  | **EBV+ n=44419** | **EBV- n=1013** |
| ebv-miR-BART7 | 241 | 0,54 | 23,79 |
| ebv-miR-BART5 | 108 | 0,24 | 10,66 |
| ebv-miR-BART11-5p | 63 | 0,14 | 6,22 |
| ebv-miR-BART1-5p | 49 | 0,11 | 4,84 |
| ebv-miR-BART19-3p | 48 | 0,11 | 4,74 |
| ebv-miR-BART14 | 37 | 0,08 | 3,65 |
| ebv-miR-BART16 | 37 | 0,08 | 3,65 |
| ebv-miR-BART9 | 34 | 0,08 | 3,36 |
| ebv-miR-BART22 | 34 | 0,08 | 3,36 |
| ebv-miR-BART2-5p | 31 | 0,07 | 3,06 |
| ebv-miR-BART13 | 30 | 0,07 | 2,96 |
| ebv-miR-BART17-5p | 29 | 0,07 | 2,86 |
| ebv-miR-BART1-3p | 28 | 0,06 | 2,76 |
| ebv-miR-BART4 | 26 | 0,06 | 2,57 |
| ebv-miR-BART11-3p | 26 | 0,06 | 2,57 |
| ebv-miR-BART10 | 25 | 0,06 | 2,47 |
| ebv-miR-BART17-3p | 24 | 0,05 | 2,37 |
| ebv-miR-BART3 | 18 | 0,04 | 1,78 |
| ebv-miR-BART8* | 18 | 0,04 | 1,78 |
| ebv-miR-BART6-5p | 17 | 0,04 | 1,68 |
| ebv-miR-BART6-3p | 15 | 0,03 | 1,48 |
| ebv-miR-BART8 | 10 | 0,02 | 0,99 |
| ebv-miR-BART3* | 9 | 0,02 | 0,89 |
| ebv-miR-BART4* | 8 | 0,02 | 0,79 |
| ebv-miR-BART12 | 7 | 0,02 | 0,69 |
| ebv-miR-BART20-3p | 7 | 0,02 | 0,69 |
| ebv-miR-BART21-3p | 7 | 0,02 | 0,69 |
| ebv-miR-BART21-5p | 6 | 0,01 | 0,59 |
| ebv-miR-BART2-3p | 5 | 0,01 | 0,49 |
| ebv-miR-BART19-5p | 5 | 0,01 | 0,49 |
| ebv-miR-BART20-5p | 3 | 0,01 | 0,30 |
| ebv-miR-BART9* | 2 | 0,00 | 0,20 |
| ebv-miR-BART15 | 2 | 0,00 | 0,20 |
| ebv-miR-BART18-5p | 2 | 0,00 | 0,20 |
| ebv-miR-BART7* | 1 | 0,00 | 0,10 |
| ebv-miR-BART14* | 1 | 0,00 | 0,10 |
| ebv-miR-BHRF1-1 | 0 | 0,00 | 0,00 |
| ebv-miR-BHRF1-2 | 0 | 0,00 | 0,00 |
| ebv-miR-BHRF1-2* | 0 | 0,00 | 0,00 |
