# Supplementary material for: MicroRNA Profiling of Epstein-Barr Virus-Associated NK/T-Cell Lymphomas by Deep Sequencing
Source: PLoS One. 2012 Aug 3;7(8):e42193. doi: 10.1371/journal.pone.0042193 (PMC3411711; doi:10.1371/journal.pone.0042193)
Supplement: Table S2 — Differentially expressed miRNAs in EBV-negative T-cell lymphoma compared to Thymus. (DOC) [file pone.0042193.s008.doc]

**Supporting Table S2**

| **miRNA** | **relative expression change EBV-/Thymus** | **rel.miRNA Expression [%]** | **rel.miRNA Expression [%]** |
| --- | --- | --- | --- |
|  |  | **Thymus** | **EBV-** |
| hsa-let-7f | 7,26 | 0,06 | 0,41 |
| hsa-miR-21 | 6,10 | 1,47 | 8,97 |
| hsa-miR-155 | 5,98 | 0,21 | 1,25 |
| hsa-miR-142-5p | 4,51 | 0,89 | 4,02 |
| hsa-miR-34a | 3,98 | 0,14 | 0,57 |
| hsa-miR-150 | 3,45 | 0,23 | 0,80 |
| hsa-miR-32 | 3,15 | 0,05 | 0,16 |
| hsa-miR-374a | 3,04 | 0,09 | 0,28 |
| hsa-let-7a | 2,73 | 0,12 | 0,33 |
| hsa-miR-146a+146b-5p | 2,68 | 0,69 | 1,85 |
| hsa-miR-26b | 2,61 | 0,84 | 2,19 |
| hsa-let-7g | 2,26 | 0,19 | 0,44 |
| hsa-miR-142-3p | 2,18 | 2,34 | 5,10 |
| hsa-miR-20b | 1,98 | 0,41 | 0,84 |
| hsa-miR-1280 | 0,54 | 0,09 | 0,05 |
| hsa-miR-27b | 0,53 | 1,83 | 0,97 |
| hsa-miR-30e | 0,52 | 0,67 | 0,35 |
| hsa-miR-186 | 0,52 | 0,12 | 0,06 |
| hsa-miR-30d | 0,52 | 0,22 | 0,11 |
| hsa-miR-195 | 0,50 | 1,30 | 0,66 |
| hsa-miR-342-3p | 0,50 | 0,60 | 0,30 |
| hsa-miR-140-5p | 0,49 | 0,14 | 0,07 |
| hsa-miR-30a | 0,48 | 0,10 | 0,05 |
| hsa-miR-484 | 0,46 | 0,27 | 0,12 |
| hsa-miR-126 | 0,46 | 3,53 | 1,62 |
| hsa-miR-22* | 0,44 | 0,13 | 0,06 |
| hsa-miR-22 | 0,43 | 0,99 | 0,42 |
| hsa-miR-148a | 0,42 | 0,27 | 0,11 |
| hsa-miR-145 | 0,41 | 0,51 | 0,21 |
| hsa-miR-152 | 0,40 | 0,32 | 0,13 |
| hsa-miR-151-3p | 0,40 | 0,14 | 0,06 |
| hsa-miR-425 | 0,38 | 0,44 | 0,17 |
| hsa-miR-497 | 0,36 | 1,24 | 0,44 |
| hsa-miR-320 | 0,34 | 1,29 | 0,44 |
| hsa-miR-128a+128b | 0,31 | 0,86 | 0,26 |
| hsa-miR-190 | 0,31 | 0,23 | 0,07 |
| hsa-miR-125a-5p | 0,30 | 0,21 | 0,06 |
| hsa-miR-29c | 0,30 | 1,01 | 0,30 |
| hsa-miR-424 | 0,27 | 2,51 | 0,67 |
| hsa-miR-378 | 0,23 | 0,28 | 0,06 |
| hsa-miR-151-5p | 0,22 | 1,25 | 0,27 |
| hsa-miR-455-3p | 0,20 | 0,77 | 0,15 |
| hsa-miR-143 | 0,18 | 0,53 | 0,09 |
| hsa-miR-218 | 0,09 | 0,50 | 0,05 |
| hsa-miR-125b | 0,06 | 1,45 | 0,08 |
