# Supplementary material for: MicroRNA Profiling of Epstein-Barr Virus-Associated NK/T-Cell Lymphomas by Deep Sequencing
Source: PLoS One. 2012 Aug 3;7(8):e42193. doi: 10.1371/journal.pone.0042193 (PMC3411711; doi:10.1371/journal.pone.0042193)
Supplement: Table S3 — Differentially expressed miRNAs in EBV-positive T-cell lymphoma compared to Thymus. (DOC) [file pone.0042193.s009.doc]

**Supporting Table S3**

| **miRNA** | **relative expression change EBV+/Thymus** | **rel.miRNA Expression [%]** | **rel.miRNA Expression [%]** |
| --- | --- | --- | --- |
|  |  | **Thymus** | **EBV+** |
| hsa-miR-155 | 6,83 | 0,21 | 1,43 |
| hsa-let-7f | 6,76 | 0,06 | 0,38 |
| hsa-miR-145 | 4,51 | 0,51 | 2,31 |
| hsa-miR-21 | 3,99 | 1,47 | 5,87 |
| hsa-let-7a | 3,83 | 0,12 | 0,45 |
| hsa-miR-26b | 3,46 | 0,84 | 2,90 |
| hsa-miR-29b | 3,18 | 0,18 | 0,58 |
| hsa-let-7g | 3,17 | 0,19 | 0,61 |
| hsa-miR-150 | 3,15 | 0,23 | 0,73 |
| hsa-miR-34a | 3,08 | 0,14 | 0,44 |
| hsa-miR-32 | 2,92 | 0,05 | 0,15 |
| hsa-miR-1280 | 2,80 | 0,09 | 0,25 |
| hsa-let-7b | 2,65 | 0,11 | 0,28 |
| hsa-miR-30a | 2,51 | 0,10 | 0,24 |
| hsa-miR-26a | 2,32 | 1,94 | 4,52 |
| hsa-miR-142-5p | 2,30 | 0,89 | 2,05 |
| hsa-miR-374a | 2,26 | 0,09 | 0,21 |
| hsa-miR-214 | 2,16 | 0,05 | 0,11 |
| hsa-miR-342-3p | 0,54 | 0,60 | 0,32 |
| hsa-miR-22 | 0,53 | 0,99 | 0,53 |
| hsa-miR-191 | 0,52 | 1,27 | 0,66 |
| hsa-miR-320 | 0,48 | 1,29 | 0,62 |
| hsa-miR-425 | 0,45 | 0,44 | 0,20 |
| hsa-miR-429 | 0,44 | 0,15 | 0,07 |
| hsa-miR-25 | 0,43 | 1,26 | 0,55 |
| hsa-miR-125b | 0,39 | 1,45 | 0,57 |
| hsa-miR-152 | 0,38 | 0,32 | 0,12 |
| hsa-miR-106b | 0,38 | 2,21 | 0,84 |
| hsa-miR-19b | 0,37 | 1,18 | 0,44 |
| hsa-miR-200a | 0,34 | 0,26 | 0,09 |
| hsa-miR-106a+17 | 0,34 | 3,38 | 1,16 |
| hsa-miR-99a | 0,29 | 0,24 | 0,07 |
| hsa-miR-484 | 0,28 | 0,27 | 0,08 |
| hsa-miR-17* | 0,25 | 0,33 | 0,08 |
| hsa-miR-190 | 0,23 | 0,23 | 0,05 |
| hsa-miR-181a | 0,21 | 1,27 | 0,26 |
| hsa-miR-451 | 0,20 | 0,32 | 0,06 |
| hsa-miR-205 | 0,19 | 1,49 | 0,28 |
| hsa-miR-20b | 0,18 | 0,41 | 0,07 |
| hsa-miR-378 | 0,16 | 0,28 | 0,05 |
| hsa-miR-181b | 0,15 | 0,58 | 0,41 |
| hsa-miR-455-3p | 0,14 | 0,77 | 0,11 |
| hsa-miR-100 | 0,13 | 0,45 | 0,06 |
| hsa-miR-218 | 0,12 | 0,50 | 0,06 |
| hsa-miR-424 | 0,11 | 2,51 | 0,28 |
| hsa-miR-128a+128b | 0,09 | 0,86 | 0,07 |
