# Supplementary material for: MicroRNA Profiling of Epstein-Barr Virus-Associated NK/T-Cell Lymphomas by Deep Sequencing
Source: PLoS One. 2012 Aug 3;7(8):e42193. doi: 10.1371/journal.pone.0042193 (PMC3411711; doi:10.1371/journal.pone.0042193)
Supplement: Table S4 — Absolute and relative miRNA expression in the small RNA libraries analysed by sequencing. (DOC) [file pone.0042193.s010.doc]

**Supporting Table S4**

| **miRNA** | **total reads** | **rel.miRNA expression [%]** | **total reads** | **rel.miRNA expression [%]** | **total reads** | **rel.miRNA expression [%]** |
| --- | --- | --- | --- | --- | --- | --- |
|  | **Thymus n=31961** | | **EBV- n=29837** | | **EBV+ n=44419** | |
| hsa-let-7a | 38 | 0,12 | 97 | 0,33 | 202 | 0,45 |
| hsa-let-7a* | 13 | 0,04 | 3 | 0,01 | 10 | 0,02 |
| hsa-let-7a-2* | 3 | 0,01 | 0 | 0,00 | 0 | 0,00 |
| hsa-let-7b | 34 | 0,11 | 32 | 0,11 | 125 | 0,28 |
| hsa-let-7b* | 1 | 0,00 | 0 | 0,00 | 6 | 0,01 |
| hsa-let-7c | 18 | 0,06 | 12 | 0,04 | 41 | 0,09 |
| hsa-let-7c-3p | 2 | 0,01 | 0 | 0,00 | 0 | 0,00 |
| hsa-let-7d | 8 | 0,03 | 20 | 0,07 | 21 | 0,05 |
| hsa-let-7d* | 6 | 0,02 | 4 | 0,01 | 11 | 0,02 |
| hsa-let-7e | 2 | 0,01 | 4 | 0,01 | 9 | 0,02 |
| hsa-let-7e* | 2 | 0,01 | 1 | 0,00 | 0 | 0,00 |
| hsa-let-7f | 18 | 0,06 | 122 | 0,41 | 169 | 0,38 |
| hsa-let-7f-1* | 4 | 0,01 | 1 | 0,00 | 4 | 0,01 |
| hsa-let-7f-2* | 5 | 0,02 | 6 | 0,02 | 6 | 0,01 |
| hsa-let-7g | 62 | 0,19 | 131 | 0,44 | 273 | 0,61 |
| hsa-let-7g* | 16 | 0,05 | 8 | 0,03 | 8 | 0,02 |
| hsa-let-7i | 22 | 0,07 | 19 | 0,06 | 45 | 0,10 |
| hsa-let-7i* | 25 | 0,08 | 22 | 0,07 | 22 | 0,05 |
| hsa-miR-1 | 1 | 0,00 | 2 | 0,01 | 2 | 0,00 |
| hsa-miR-100 | 145 | 0,45 | 4 | 0,01 | 27 | 0,06 |
| hsa-miR-100* | 2 | 0,01 | 0 | 0,00 | 0 | 0,00 |
| hsa-miR-101 | 344 | 1,08 | 291 | 0,98 | 298 | 0,67 |
| hsa-miR-101* | 1 | 0,00 | 0 | 0,00 | 2 | 0,00 |
| hsa-miR-103+107 | 902 | 2,82 | 738 | 2,47 | 825 | 1,86 |
| hsa-miR-103-5p | 22 | 0,07 | 12 | 0,04 | 17 | 0,04 |
| hsa-miR-106a+17 | 1081 | 3,38 | 1090 | 3,65 | 514 | 1,16 |
| hsa-miR-106a* | 0 | 0,00 | 1 | 0,00 | 0 | 0,00 |
| hsa-miR-106b | 706 | 2,21 | 528 | 1,77 | 374 | 0,84 |
| hsa-miR-106b* | 7 | 0,02 | 7 | 0,02 | 10 | 0,02 |
| hsa-miR-107-5p | 3 | 0,01 | 0 | 0,00 | 1 | 0,00 |
| hsa-miR-10a | 6 | 0,02 | 6 | 0,02 | 0 | 0,00 |
| hsa-miR-10b | 38 | 0,12 | 5 | 0,02 | 15 | 0,03 |
| hsa-miR-10b* | 0 | 0,00 | 0 | 0,00 | 1 | 0,00 |
| hsa-miR-1185-3p | 2 | 0,01 | 0 | 0,00 | 0 | 0,00 |
| hsa-miR-1248 | 0 | 0,00 | 0 | 0,00 | 1 | 0,00 |
| hsa-miR-1248a-3p | 0 | 0,00 | 0 | 0,00 | 4 | 0,01 |
| hsa-miR-1249 | 2 | 0,01 | 0 | 0,00 | 0 | 0,00 |
| hsa-miR-125a-3p | 1 | 0,00 | 0 | 0,00 | 0 | 0,00 |
| hsa-miR-125a-5p | 67 | 0,21 | 19 | 0,06 | 108 | 0,24 |
| hsa-miR-125b | 464 | 1,45 | 25 | 0,08 | 253 | 0,57 |
| hsa-miR-125b* | 5 | 0,02 | 0 | 0,00 | 0 | 0,00 |
| hsa-miR-125b-1* | 0 | 0,00 | 0 | 0,00 | 1 | 0,00 |
| hsa-miR-125b-2 | 0 | 0,00 | 0 | 0,00 | 4 | 0,01 |
| hsa-miR-125b-2* | 0 | 0,00 | 0 | 0,00 | 1 | 0,00 |
| hsa-miR-126 | 1129 | 3,53 | 482 | 1,62 | 880 | 1,98 |
| hsa-miR-126* | 142 | 0,44 | 113 | 0,38 | 316 | 0,71 |
| hsa-miR-1261 | 2 | 0,01 | 0 | 0,00 | 0 | 0,00 |
| hsa-miR-1268 | 2 | 0,01 | 0 | 0,00 | 0 | 0,00 |
| hsa-miR-1271 | 3 | 0,01 | 0 | 0,00 | 0 | 0,00 |
| hsa-miR-1273 | 0 | 0,00 | 0 | 0,00 | 2 | 0,00 |
| hsa-miR-127-3p | 10 | 0,03 | 2 | 0,01 | 0 | 0,00 |
| hsa-miR-1274b | 4 | 0,01 | 0 | 0,00 | 12 | 0,03 |
| hsa-miR-1275 | 0 | 0,00 | 0 | 0,00 | 2 | 0,00 |
| hsa-miR-127-5p | 6 | 0,02 | 3 | 0,01 | 0 | 0,00 |
| hsa-miR-1277-5p | 0 | 0,00 | 3 | 0,01 | 0 | 0,00 |
| hsa-miR-1280 | 28 | 0,09 | 14 | 0,05 | 109 | 0,25 |
| hsa-miR-128a+128b | 274 | 0,86 | 79 | 0,26 | 33 | 0,07 |
| hsa-miR-1301 | 6 | 0,02 | 5 | 0,02 | 0 | 0,00 |
| hsa-miR-1307 | 7 | 0,02 | 3 | 0,01 | 4 | 0,01 |
| hsa-miR-1308 | 0 | 0,00 | 4 | 0,01 | 0 | 0,00 |
| hsa-miR-130a | 103 | 0,32 | 53 | 0,18 | 122 | 0,27 |
| hsa-miR-130b | 13 | 0,04 | 17 | 0,06 | 16 | 0,04 |
| hsa-miR-130b* | 1 | 0,00 | 1 | 0,00 | 1 | 0,00 |
| hsa-miR-132 | 26 | 0,08 | 4 | 0,01 | 9 | 0,02 |
| hsa-miR-132* | 2 | 0,01 | 0 | 0,00 | 0 | 0,00 |
| hsa-miR-133a+133b | 20 | 0,06 | 0 | 0,00 | 2 | 0,00 |
| hsa-miR-134 | 13 | 0,04 | 0 | 0,00 | 5 | 0,01 |
| hsa-miR-135a | 4 | 0,01 | 2 | 0,01 | 59 | 0,13 |
| hsa-miR-135b | 3 | 0,01 | 27 | 0,09 | 16 | 0,04 |
| hsa-miR-135b* | 0 | 0,00 | 0 | 0,00 | 1 | 0,00 |
| hsa-miR-136 | 3 | 0,01 | 0 | 0,00 | 1 | 0,00 |
| hsa-miR-136* | 13 | 0,04 | 0 | 0,00 | 1 | 0,00 |
| hsa-miR-137 | 1 | 0,00 | 0 | 0,00 | 0 | 0,00 |
| hsa-miR-138 | 1 | 0,00 | 5 | 0,02 | 9 | 0,02 |
| hsa-miR-138-1* | 0 | 0,00 | 0 | 0,00 | 1 | 0,00 |
| hsa-miR-139-5p | 31 | 0,10 | 20 | 0,07 | 26 | 0,06 |
| hsa-miR-140-3p | 52 | 0,16 | 76 | 0,25 | 137 | 0,31 |
| hsa-miR-140-5p | 44 | 0,14 | 20 | 0,07 | 57 | 0,13 |
| hsa-miR-141 | 114 | 0,36 | 2 | 0,01 | 94 | 0,21 |
| hsa-miR-141* | 2 | 0,01 | 0 | 0,00 | 1 | 0,00 |
| hsa-miR-142-3p | 748 | 2,34 | 1522 | 5,10 | 860 | 1,94 |
| hsa-miR-142-5p | 285 | 0,89 | 1199 | 4,02 | 909 | 2,05 |
| hsa-miR-143 | 170 | 0,53 | 28 | 0,09 | 320 | 0,72 |
| hsa-miR-143* | 1 | 0,00 | 2 | 0,01 | 2 | 0,00 |
| hsa-miR-144 | 8 | 0,03 | 7 | 0,02 | 5 | 0,01 |
| hsa-miR-145 | 164 | 0,51 | 63 | 0,21 | 1028 | 2,31 |
| hsa-miR-145* | 7 | 0,02 | 0 | 0,00 | 3 | 0,01 |
| hsa-miR-146a+146b-5p | 221 | 0,69 | 552 | 1,85 | 176 | 0,40 |
| hsa-miR-146b-3p | 0 | 0,00 | 0 | 0,00 | 1 | 0,00 |
| hsa-miR-148a | 85 | 0,27 | 33 | 0,11 | 205 | 0,46 |
| hsa-miR-148a* | 2 | 0,01 | 0 | 0,00 | 2 | 0,00 |
| hsa-miR-148b | 24 | 0,08 | 11 | 0,04 | 8 | 0,02 |
| hsa-miR-148b* | 0 | 0,00 | 0 | 0,00 | 1 | 0,00 |
| hsa-miR-149 | 17 | 0,05 | 0 | 0,00 | 0 | 0,00 |
| hsa-miR-150 | 74 | 0,23 | 238 | 0,80 | 324 | 0,73 |
| hsa-miR-150* | 3 | 0,01 | 1 | 0,00 | 6 | 0,01 |
| hsa-miR-151-3p | 46 | 0,14 | 17 | 0,06 | 46 | 0,10 |
| hsa-miR-151-5p | 399 | 1,25 | 82 | 0,27 | 315 | 0,71 |
| hsa-miR-152 | 101 | 0,32 | 38 | 0,13 | 54 | 0,12 |
| hsa-miR-153 | 0 | 0,00 | 15 | 0,05 | 2 | 0,00 |
| hsa-miR-153-2-5p | 0 | 0,00 | 4 | 0,01 | 0 | 0,00 |
| hsa-miR-154 | 6 | 0,02 | 2 | 0,01 | 1 | 0,00 |
| hsa-miR-154* | 2 | 0,01 | 1 | 0,00 | 0 | 0,00 |
| hsa-miR-155 | 67 | 0,21 | 374 | 1,25 | 636 | 1,43 |
| hsa-miR-155* | 0 | 0,00 | 0 | 0,00 | 2 | 0,00 |
| hsa-miR-15a | 1268 | 3,97 | 1024 | 3,43 | 1315 | 2,96 |
| hsa-miR-15a* | 9 | 0,03 | 6 | 0,02 | 5 | 0,01 |
| hsa-miR-15b | 889 | 2,78 | 690 | 2,31 | 810 | 1,82 |
| hsa-miR-15b* | 3 | 0,01 | 3 | 0,01 | 0 | 0,00 |
| hsa-miR-16 | 2266 | 7,09 | 3607 | 12,09 | 4036 | 9,09 |
| hsa-miR-16-1* | 1 | 0,00 | 1 | 0,00 | 0 | 0,00 |
| hsa-miR-16-2 | 0 | 0,00 | 0 | 0,00 | 4 | 0,01 |
| hsa-miR-16-2* | 24 | 0,08 | 15 | 0,05 | 13 | 0,03 |
| hsa-miR-163 | 0 | 0,00 | 0 | 0,00 | 4 | 0,01 |
| hsa-miR-17* | 107 | 0,33 | 92 | 0,31 | 37 | 0,08 |
| hsa-miR-181a | 405 | 1,27 | 265 | 0,89 | 116 | 0,26 |
| hsa-miR-181a* | 2 | 0,01 | 6 | 0,02 | 0 | 0,00 |
| hsa-miR-181a-2* | 1 | 0,00 | 4 | 0,01 | 0 | 0,00 |
| hsa-miR-181b | 186 | 0,58 | 121 | 0,41 | 38 | 0,09 |
| hsa-miR-181c | 4 | 0,01 | 2 | 0,01 | 3 | 0,01 |
| hsa-miR-181c* | 1 | 0,00 | 0 | 0,00 | 0 | 0,00 |
| hsa-miR-181d | 5 | 0,02 | 2 | 0,01 | 3 | 0,01 |
| hsa-miR-182 | 53 | 0,17 | 4 | 0,01 | 7 | 0,02 |
| hsa-miR-183 | 22 | 0,07 | 1 | 0,00 | 2 | 0,00 |
| hsa-miR-184 | 0 | 0,00 | 0 | 0,00 | 4 | 0,01 |
| hsa-miR-185 | 58 | 0,18 | 89 | 0,30 | 8 | 0,02 |
| hsa-miR-185* | 0 | 0,00 | 1 | 0,00 | 0 | 0,00 |
| hsa-miR-186 | 37 | 0,12 | 18 | 0,06 | 36 | 0,08 |
| hsa-miR-187 | 2 | 0,01 | 0 | 0,00 | 1 | 0,00 |
| hsa-miR-188-3p | 0 | 0,00 | 0 | 0,00 | 1 | 0,00 |
| hsa-miR-188-5p | 0 | 0,00 | 0 | 0,00 | 1 | 0,00 |
| hsa-miR-18a+b | 43 | 0,13 | 54 | 0,18 | 16 | 0,04 |
| hsa-miR-18a* | 1 | 0,00 | 2 | 0,01 | 2 | 0,00 |
| hsa-miR-190 | 73 | 0,23 | 21 | 0,07 | 23 | 0,05 |
| hsa-miR-190b | 3 | 0,01 | 4 | 0,01 | 9 | 0,02 |
| hsa-miR-191 | 407 | 1,27 | 258 | 0,86 | 295 | 0,66 |
| hsa-miR-192 | 15 | 0,05 | 1 | 0,00 | 2 | 0,00 |
| hsa-miR-192* | 2 | 0,01 | 1 | 0,00 | 0 | 0,00 |
| hsa-miR-193a-3p | 7 | 0,02 | 5 | 0,02 | 10 | 0,02 |
| hsa-miR-193a-5p | 1 | 0,00 | 1 | 0,00 | 2 | 0,00 |
| hsa-miR-193b | 16 | 0,05 | 4 | 0,01 | 18 | 0,04 |
| hsa-miR-193b* | 1 | 0,00 | 0 | 0,00 | 0 | 0,00 |
| hsa-miR-194 | 22 | 0,07 | 8 | 0,03 | 12 | 0,03 |
| hsa-miR-195 | 416 | 1,30 | 196 | 0,66 | 886 | 1,99 |
| hsa-miR-196a | 0 | 0,00 | 10 | 0,03 | 0 | 0,00 |
| hsa-miR-196b | 5 | 0,02 | 3 | 0,01 | 0 | 0,00 |
| hsa-miR-196b* | 0 | 0,00 | 1 | 0,00 | 0 | 0,00 |
| hsa-miR-197 | 2 | 0,01 | 0 | 0,00 | 6 | 0,01 |
| hsa-miR-199a-3+199b-3p | 1223 | 3,83 | 1328 | 4,45 | 1814 | 4,08 |
| hsa-miR-199a-5p | 242 | 0,76 | 268 | 0,90 | 419 | 0,94 |
| hsa-miR-199b-5p | 82 | 0,26 | 7 | 0,02 | 97 | 0,22 |
| hsa-miR-19a | 27 | 0,08 | 34 | 0,11 | 23 | 0,05 |
| hsa-miR-19b | 378 | 1,18 | 261 | 0,87 | 196 | 0,44 |
| hsa-mir-19b* | 0 | 0,00 | 2 | 0,01 | 0 | 0,00 |
| hsa-miR-19b-2* | 1 | 0,00 | 0 | 0,00 | 0 | 0,00 |
| hsa-miR-200a | 84 | 0,26 | 0 | 0,00 | 40 | 0,09 |
| hsa-miR-200a* | 4 | 0,01 | 0 | 0,00 | 1 | 0,00 |
| hsa-miR-200b | 692 | 2,17 | 0 | 0,00 | 525 | 1,18 |
| hsa-miR-200b* | 7 | 0,02 | 0 | 0,00 | 5 | 0,01 |
| hsa-miR-200c | 282 | 0,88 | 9 | 0,03 | 458 | 1,03 |
| hsa-miR-200c* | 1 | 0,00 | 0 | 0,00 | 0 | 0,00 |
| hsa-miR-202* | 0 | 0,00 | 0 | 0,00 | 2 | 0,00 |
| hsa-miR-203 | 36 | 0,11 | 0 | 0,00 | 43 | 0,10 |
| hsa-miR-204 | 37 | 0,12 | 0 | 0,00 | 30 | 0,07 |
| hsa-miR-205 | 476 | 1,49 | 0 | 0,00 | 123 | 0,28 |
| hsa-miR-205* | 10 | 0,03 | 0 | 0,00 | 2 | 0,00 |
| hsa-miR-206 | 13 | 0,04 | 0 | 0,00 | 0 | 0,00 |
| hsa-miR-208b | 2 | 0,01 | 0 | 0,00 | 0 | 0,00 |
| hsa-miR-20a | 857 | 2,68 | 974 | 3,26 | 698 | 1,57 |
| hsa-miR-20a* | 4 | 0,01 | 3 | 0,01 | 1 | 0,00 |
| hsa-miR-20b | 131 | 0,41 | 242 | 0,81 | 32 | 0,07 |
| hsa-miR-20b* | 0 | 0,00 | 5 | 0,02 | 0 | 0,00 |
| hsa-miR-21 | 470 | 1,47 | 2676 | 8,97 | 2605 | 5,86 |
| hsa-miR-21* | 9 | 0,03 | 31 | 0,10 | 16 | 0,04 |
| hsa-miR-210 | 8 | 0,03 | 3 | 0,01 | 17 | 0,04 |
| hsa-miR-212 | 5 | 0,02 | 3 | 0,01 | 0 | 0,00 |
| hsa-miR-214 | 16 | 0,05 | 27 | 0,09 | 48 | 0,11 |
| hsa-miR-214* | 2 | 0,01 | 0 | 0,00 | 1 | 0,00 |
| hsa-miR-216a | 0 | 0,00 | 0 | 0,00 | 1 | 0,00 |
| hsa-miR-218 | 160 | 0,50 | 14 | 0,05 | 27 | 0,06 |
| hsa-miR-219-5p | 3 | 0,01 | 6 | 0,02 | 2 | 0,00 |
| hsa-miR-22 | 315 | 0,99 | 126 | 0,42 | 234 | 0,53 |
| hsa-miR-22* | 41 | 0,13 | 17 | 0,06 | 42 | 0,09 |
| hsa-miR-221 | 336 | 1,05 | 324 | 1,09 | 409 | 0,92 |
| hsa-miR-221* | 1 | 0,00 | 8 | 0,03 | 13 | 0,03 |
| hsa-miR-2210 | 0 | 0,00 | 0 | 0,00 | 4 | 0,01 |
| hsa-miR-222 | 71 | 0,22 | 90 | 0,30 | 154 | 0,35 |
| hsa-miR-223 | 199 | 0,62 | 209 | 0,70 | 260 | 0,59 |
| hsa-miR-223* | 2 | 0,01 | 1 | 0,00 | 19 | 0,04 |
| hsa-miR-224 | 7 | 0,02 | 6 | 0,02 | 6 | 0,01 |
| hsa-miR-23b+23a | 1444 | 4,52 | 1389 | 4,66 | 3209 | 7,22 |
| hsa-miR-23b* | 1 | 0,00 | 0 | 0,00 | 0 | 0,00 |
| hsa-miR-24 | 290 | 0,91 | 226 | 0,76 | 455 | 1,02 |
| hsa-miR-24-1* | 0 | 0,00 | 0 | 0,00 | 3 | 0,01 |
| hsa-miR-24-2* | 7 | 0,02 | 0 | 0,00 | 3 | 0,01 |
| hsa-miR-25 | 404 | 1,26 | 287 | 0,96 | 244 | 0,55 |
| hsa-miR-26a | 621 | 1,94 | 819 | 2,74 | 2006 | 4,52 |
| hsa-miR-26a-1* | 1 | 0,00 | 0 | 0,00 | 0 | 0,00 |
| hsa-miR-26b | 268 | 0,84 | 652 | 2,19 | 1289 | 2,90 |
| hsa-miR-26b* | 1 | 0,00 | 0 | 0,00 | 4 | 0,01 |
| hsa-miR-27a | 1096 | 3,43 | 1309 | 4,39 | 2224 | 5,01 |
| hsa-miR-27b | 585 | 1,83 | 288 | 0,97 | 1468 | 3,30 |
| hsa-miR-27b* | 1 | 0,00 | 0 | 0,00 | 1 | 0,00 |
| hsa-miR-28-3p | 37 | 0,12 | 24 | 0,08 | 46 | 0,10 |
| hsa-miR-28-5p | 81 | 0,25 | 58 | 0,19 | 100 | 0,23 |
| hsa-miR-29a | 281 | 0,88 | 234 | 0,78 | 670 | 1,51 |
| hsa-miR-29a* | 5 | 0,02 | 1 | 0,00 | 6 | 0,01 |
| hsa-miR-29b | 58 | 0,18 | 65 | 0,22 | 256 | 0,58 |
| hsa-miR-29b-1* | 0 | 0,00 | 1 | 0,00 | 1 | 0,00 |
| hsa-miR-29b-2* | 2 | 0,01 | 1 | 0,00 | 2 | 0,00 |
| hsa-miR-29c | 324 | 1,01 | 90 | 0,30 | 537 | 1,21 |
| hsa-miR-29c* | 3 | 0,01 | 2 | 0,01 | 2 | 0,00 |
| hsa-miR-301a | 22 | 0,07 | 24 | 0,08 | 20 | 0,05 |
| hsa-miR-30a | 31 | 0,10 | 14 | 0,05 | 108 | 0,24 |
| hsa-miR-30a* | 13 | 0,04 | 1 | 0,00 | 18 | 0,04 |
| hsa-miR-30b | 327 | 1,02 | 194 | 0,65 | 375 | 0,84 |
| hsa-miR-30c | 173 | 0,54 | 138 | 0,46 | 238 | 0,54 |
| hsa-miR-30c-2* | 0 | 0,00 | 0 | 0,00 | 1 | 0,00 |
| hsa-miR-30d | 70 | 0,22 | 34 | 0,11 | 135 | 0,30 |
| hsa-miR-30d* | 6 | 0,02 | 0 | 0,00 | 0 | 0,00 |
| hsa-miR-30e | 213 | 0,67 | 104 | 0,35 | 195 | 0,44 |
| hsa-miR-30e* | 42 | 0,13 | 38 | 0,13 | 52 | 0,12 |
| hsa-miR-31 | 7 | 0,02 | 4 | 0,01 | 21 | 0,05 |
| hsa-miR-31* | 9 | 0,03 | 0 | 0,00 | 12 | 0,03 |
| hsa-miR-32 | 16 | 0,05 | 47 | 0,16 | 65 | 0,15 |
| hsa-miR-32* | 0 | 0,00 | 4 | 0,01 | 2 | 0,00 |
| hsa-miR-320 | 413 | 1,29 | 130 | 0,44 | 275 | 0,62 |
| hsa-miR-323-3p | 5 | 0,02 | 1 | 0,00 | 2 | 0,00 |
| hsa-miR-324-3p | 23 | 0,07 | 7 | 0,02 | 14 | 0,03 |
| hsa-miR-324-5p | 15 | 0,05 | 5 | 0,02 | 7 | 0,02 |
| hsa-miR-326 | 1 | 0,00 | 0 | 0,00 | 2 | 0,00 |
| hsa-miR-328 | 4 | 0,01 | 0 | 0,00 | 2 | 0,00 |
| hsa-miR-329 | 4 | 0,01 | 0 | 0,00 | 0 | 0,00 |
| hsa-miR-330-3p | 1 | 0,00 | 1 | 0,00 | 2 | 0,00 |
| hsa-miR-331 | 2 | 0,01 | 0 | 0,00 | 2 | 0,00 |
| hsa-miR-331-3p | 0 | 0,00 | 7 | 0,02 | 6 | 0,01 |
| hsa-miR-335 | 21 | 0,07 | 6 | 0,02 | 11 | 0,02 |
| hsa-miR-335* | 1 | 0,00 | 0 | 0,00 | 0 | 0,00 |
| hsa-miR-337-3p | 13 | 0,04 | 0 | 0,00 | 6 | 0,01 |
| hsa-miR-337-5p | 6 | 0,02 | 0 | 0,00 | 1 | 0,00 |
| hsa-miR-338-3p | 3 | 0,01 | 0 | 0,00 | 6 | 0,01 |
| hsa-miR-338-5p | 0 | 0,00 | 0 | 0,00 | 1 | 0,00 |
| hsa-miR-339-3p | 6 | 0,02 | 0 | 0,00 | 7 | 0,02 |
| hsa-miR-339-5p | 0 | 0,00 | 1 | 0,00 | 7 | 0,02 |
| hsa-miR-33a | 0 | 0,00 | 0 | 0,00 | 1 | 0,00 |
| hsa-miR-33a* | 2 | 0,01 | 2 | 0,01 | 1 | 0,00 |
| hsa-miR-340 | 8 | 0,03 | 7 | 0,02 | 12 | 0,03 |
| hsa-miR-340* | 1 | 0,00 | 2 | 0,01 | 2 | 0,00 |
| hsa-miR-342-3p | 192 | 0,60 | 90 | 0,30 | 143 | 0,32 |
| hsa-miR-342-5p | 1 | 0,00 | 0 | 0,00 | 2 | 0,00 |
| hsa-miR-345 | 21 | 0,07 | 21 | 0,07 | 18 | 0,04 |
| hsa-miR-34a | 46 | 0,14 | 171 | 0,57 | 197 | 0,44 |
| hsa-miR-34a* | 0 | 0,00 | 9 | 0,03 | 7 | 0,02 |
| hsa-miR-34b | 0 | 0,00 | 0 | 0,00 | 8 | 0,02 |
| hsa-miR-34b* | 3 | 0,01 | 2 | 0,01 | 29 | 0,07 |
| hsa-miR-34c-3p | 0 | 0,00 | 1 | 0,00 | 11 | 0,02 |
| hsa-miR-34c-5p | 2 | 0,01 | 4 | 0,01 | 64 | 0,14 |
| hsa-miR-361-3p | 2 | 0,01 | 11 | 0,04 | 14 | 0,03 |
| hsa-miR-361-5p | 61 | 0,19 | 105 | 0,35 | 99 | 0,22 |
| hsa-miR-362-3p | 13 | 0,04 | 19 | 0,06 | 11 | 0,02 |
| hsa-miR-362-5p | 8 | 0,03 | 3 | 0,01 | 6 | 0,01 |
| hsa-miR-363 | 45 | 0,14 | 59 | 0,20 | 6 | 0,01 |
| hsa-miR-365 | 38 | 0,12 | 5 | 0,02 | 19 | 0,04 |
| hsa-miR-367 | 0 | 0,00 | 0 | 0,00 | 9 | 0,02 |
| hsa-miR-369-3p | 13 | 0,04 | 3 | 0,01 | 7 | 0,02 |
| hsa-miR-369-5p | 1 | 0,00 | 0 | 0,00 | 4 | 0,01 |
| hsa-miR-370 | 1 | 0,00 | 0 | 0,00 | 1 | 0,00 |
| hsa-miR-374a | 30 | 0,09 | 85 | 0,28 | 94 | 0,21 |
| hsa-miR-374a* | 0 | 0,00 | 2 | 0,01 | 1 | 0,00 |
| hsa-miR-374b | 91 | 0,28 | 148 | 0,50 | 169 | 0,38 |
| hsa-miR-374b* | 0 | 0,00 | 2 | 0,01 | 0 | 0,00 |
| hsa-miR-375 | 5 | 0,02 | 0 | 0,00 | 12 | 0,03 |
| hsa-miR-376a | 41 | 0,13 | 0 | 0,00 | 8 | 0,02 |
| hsa-miR-376a* | 4 | 0,01 | 0 | 0,00 | 0 | 0,00 |
| hsa-miR-376a-2 | 3 | 0,01 | 0 | 0,00 | 0 | 0,00 |
| hsa-miR-376b | 2 | 0,01 | 2 | 0,01 | 4 | 0,01 |
| hsa-miR-376c | 92 | 0,29 | 3 | 0,01 | 17 | 0,04 |
| hsa-miR-377 | 9 | 0,03 | 1 | 0,00 | 3 | 0,01 |
| hsa-miR-378 | 89 | 0,28 | 19 | 0,06 | 20 | 0,05 |
| hsa-miR-378* | 10 | 0,03 | 1 | 0,00 | 2 | 0,00 |
| hsa-miR-379* | 0 | 0,00 | 0 | 0,00 | 1 | 0,00 |
| hsa-miR-381 | 6 | 0,02 | 0 | 0,00 | 0 | 0,00 |
| hsa-miR-382 | 6 | 0,02 | 1 | 0,00 | 0 | 0,00 |
| hsa-miR-382-3p | 0 | 0,00 | 2 | 0,01 | 1 | 0,00 |
| hsa-miR-383 | 1 | 0,00 | 0 | 0,00 | 0 | 0,00 |
| hsa-miR-409-3p | 3 | 0,01 | 0 | 0,00 | 0 | 0,00 |
| hsa-miR-410 | 14 | 0,04 | 0 | 0,00 | 2 | 0,00 |
| hsa-miR-411 | 1 | 0,00 | 0 | 0,00 | 0 | 0,00 |
| hsa-miR-421 | 4 | 0,01 | 10 | 0,03 | 7 | 0,02 |
| hsa-miR-423-3p | 35 | 0,11 | 13 | 0,04 | 31 | 0,07 |
| hsa-miR-423-5p | 16 | 0,05 | 3 | 0,01 | 12 | 0,03 |
| hsa-miR-424 | 802 | 2,51 | 200 | 0,67 | 123 | 0,28 |
| hsa-miR-424* | 1 | 0,00 | 1 | 0,00 | 1 | 0,00 |
| hsa-miR-425 | 141 | 0,44 | 50 | 0,17 | 89 | 0,20 |
| hsa-miR-425* | 4 | 0,01 | 3 | 0,01 | 4 | 0,01 |
| hsa-miR-429 | 47 | 0,15 | 0 | 0,00 | 29 | 0,07 |
| hsa-miR-431 | 4 | 0,01 | 0 | 0,00 | 0 | 0,00 |
| hsa-miR-432 | 9 | 0,03 | 0 | 0,00 | 3 | 0,01 |
| hsa-miR-433 | 1 | 0,00 | 0 | 0,00 | 2 | 0,00 |
| hsa-miR-449a+449b | 0 | 0,00 | 0 | 0,00 | 95 | 0,21 |
| hsa-miR-449c | 0 | 0,00 | 0 | 0,00 | 8 | 0,02 |
| hsa-miR-450a | 17 | 0,05 | 3 | 0,01 | 1 | 0,00 |
| hsa-miR-451 | 101 | 0,32 | 56 | 0,19 | 28 | 0,06 |
| hsa-miR-452 | 5 | 0,02 | 0 | 0,00 | 2 | 0,00 |
| hsa-miR-454 | 6 | 0,02 | 9 | 0,03 | 29 | 0,07 |
| hsa-miR-455-3p | 245 | 0,77 | 45 | 0,15 | 48 | 0,11 |
| hsa-miR-455-5p | 37 | 0,12 | 3 | 0,01 | 5 | 0,01 |
| hsa-miR-483-3p | 4 | 0,01 | 0 | 0,00 | 0 | 0,00 |
| hsa-miR-483-5p | 1 | 0,00 | 0 | 0,00 | 4 | 0,01 |
| hsa-miR-484 | 86 | 0,27 | 37 | 0,12 | 34 | 0,08 |
| hsa-miR-485-5p | 3 | 0,01 | 0 | 0,00 | 0 | 0,00 |
| hsa-miR-486-3p | 0 | 0,00 | 1 | 0,00 | 0 | 0,00 |
| hsa-miR-486-5p | 5 | 0,02 | 1 | 0,00 | 5 | 0,01 |
| hsa-miR-487a | 5 | 0,02 | 0 | 0,00 | 0 | 0,00 |
| hsa-miR-487b | 22 | 0,07 | 4 | 0,01 | 1 | 0,00 |
| hsa-miR-488 | 2 | 0,01 | 1 | 0,00 | 12 | 0,03 |
| hsa-miR-491-3p | 0 | 0,00 | 0 | 0,00 | 1 | 0,00 |
| hsa-miR-493 | 9 | 0,03 | 0 | 0,00 | 0 | 0,00 |
| hsa-miR-493* | 9 | 0,03 | 2 | 0,01 | 0 | 0,00 |
| hsa-miR-494 | 4 | 0,01 | 1 | 0,00 | 0 | 0,00 |
| hsa-miR-495 | 10 | 0,03 | 4 | 0,01 | 1 | 0,00 |
| hsa-miR-497 | 395 | 1,24 | 131 | 0,44 | 362 | 0,81 |
| hsa-miR-497* | 0 | 0,00 | 1 | 0,00 | 2 | 0,00 |
| hsa-miR-499-3p | 0 | 0,00 | 1 | 0,00 | 0 | 0,00 |
| hsa-miR-499-5p | 4 | 0,01 | 3 | 0,01 | 3 | 0,01 |
| hsa-miR-500 | 3 | 0,01 | 1 | 0,00 | 4 | 0,01 |
| hsa-miR-501-3p | 1 | 0,00 | 0 | 0,00 | 0 | 0,00 |
| hsa-miR-501-5p | 2 | 0,01 | 0 | 0,00 | 1 | 0,00 |
| hsa-miR-502-3p | 10 | 0,03 | 11 | 0,04 | 8 | 0,02 |
| hsa-miR-502-5p | 2 | 0,01 | 2 | 0,01 | 0 | 0,00 |
| hsa-miR-503 | 7 | 0,02 | 4 | 0,01 | 1 | 0,00 |
| hsa-miR-505 | 8 | 0,03 | 4 | 0,01 | 7 | 0,02 |
| hsa-miR-509-3-5p+509-5p | 0 | 0,00 | 3 | 0,01 | 0 | 0,00 |
| hsa-miR-511 | 3 | 0,01 | 1 | 0,00 | 2 | 0,00 |
| hsa-miR-511-3p | 0 | 0,00 | 10 | 0,03 | 4 | 0,01 |
| hsa-miR-512-5p | 0 | 0,00 | 0 | 0,00 | 1 | 0,00 |
| hsa-miR-513-5p | 0 | 0,00 | 1 | 0,00 | 0 | 0,00 |
| hsa-miR-514 | 0 | 0,00 | 1 | 0,00 | 0 | 0,00 |
| hsa-miR-520a-5p | 1 | 0,00 | 0 | 0,00 | 0 | 0,00 |
| hsa-miR-532-3p | 9 | 0,03 | 0 | 0,00 | 5 | 0,01 |
| hsa-miR-532-5p | 31 | 0,10 | 16 | 0,05 | 19 | 0,04 |
| hsa-miR-542-3p | 0 | 0,00 | 1 | 0,00 | 0 | 0,00 |
| hsa-miR-543 | 3 | 0,01 | 0 | 0,00 | 0 | 0,00 |
| hsa-miR-545* | 0 | 0,00 | 2 | 0,01 | 0 | 0,00 |
| hsa-miR-548a-3p | 0 | 0,00 | 2 | 0,01 | 3 | 0,01 |
| hsa-miR-548d-5p | 2 | 0,01 | 1 | 0,00 | 0 | 0,00 |
| hsa-miR-548h-3 | 2 | 0,01 | 0 | 0,00 | 0 | 0,00 |
| hsa-miR-551a | 1 | 0,00 | 0 | 0,00 | 0 | 0,00 |
| hsa-miR-551b | 1 | 0,00 | 0 | 0,00 | 1 | 0,00 |
| hsa-miR-566 | 3 | 0,01 | 0 | 0,00 | 0 | 0,00 |
| hsa-miR-570 | 2 | 0,01 | 2 | 0,01 | 0 | 0,00 |
| hsa-miR-574-3p | 10 | 0,03 | 5 | 0,02 | 19 | 0,04 |
| hsa-miR-574-5p | 7 | 0,02 | 1 | 0,00 | 9 | 0,02 |
| hsa-miR-576-3p | 2 | 0,01 | 0 | 0,00 | 0 | 0,00 |
| hsa-miR-576-5p | 2 | 0,01 | 0 | 0,00 | 0 | 0,00 |
| hsa-miR-582-5p | 7 | 0,02 | 0 | 0,00 | 14 | 0,03 |
| hsa-miR-584 | 0 | 0,00 | 1 | 0,00 | 1 | 0,00 |
| hsa-miR-588 | 0 | 0,00 | 0 | 0,00 | 1 | 0,00 |
| hsa-miR-589 | 1 | 0,00 | 0 | 0,00 | 1 | 0,00 |
| hsa-miR-590-3p | 7 | 0,02 | 16 | 0,05 | 51 | 0,11 |
| hsa-miR-590-5p | 6 | 0,02 | 5 | 0,02 | 5 | 0,01 |
| hsa-miR-595 | 0 | 0,00 | 0 | 0,00 | 1 | 0,00 |
| hsa-miR-598 | 25 | 0,08 | 4 | 0,01 | 3 | 0,01 |
| hsa-miR-599 | 4 | 0,01 | 0 | 0,00 | 0 | 0,00 |
| hsa-miR-610 | 1 | 0,00 | 0 | 0,00 | 0 | 0,00 |
| hsa-miR-616* | 2 | 0,01 | 0 | 0,00 | 0 | 0,00 |
| hsa-miR-620 | 1 | 0,00 | 0 | 0,00 | 0 | 0,00 |
| hsa-miR-621 | 0 | 0,00 | 0 | 0,00 | 1 | 0,00 |
| hsa-miR-624* | 1 | 0,00 | 0 | 0,00 | 5 | 0,01 |
| hsa-miR-625 | 0 | 0,00 | 4 | 0,01 | 0 | 0,00 |
| hsa-miR-625* | 0 | 0,00 | 1 | 0,00 | 0 | 0,00 |
| hsa-miR-627 | 0 | 0,00 | 1 | 0,00 | 0 | 0,00 |
| hsa-miR-628-3p | 0 | 0,00 | 3 | 0,01 | 0 | 0,00 |
| hsa-miR-628-5p | 2 | 0,01 | 0 | 0,00 | 2 | 0,00 |
| hsa-miR-629* | 0 | 0,00 | 1 | 0,00 | 3 | 0,01 |
| hsa-miR-643 | 1 | 0,00 | 4 | 0,01 | 0 | 0,00 |
| hsa-miR-645 | 0 | 0,00 | 0 | 0,00 | 1 | 0,00 |
| hsa-miR-651 | 3 | 0,01 | 0 | 0,00 | 0 | 0,00 |
| hsa-miR-652 | 42 | 0,13 | 43 | 0,14 | 33 | 0,07 |
| hsa-miR-654 | 6 | 0,02 | 0 | 0,00 | 2 | 0,00 |
| hsa-miR-655 | 4 | 0,01 | 0 | 0,00 | 0 | 0,00 |
| hsa-miR-656 | 6 | 0,02 | 2 | 0,01 | 2 | 0,00 |
| hsa-miR-660 | 7 | 0,02 | 6 | 0,02 | 7 | 0,02 |
| hsa-miR-664 | 4 | 0,01 | 0 | 0,00 | 12 | 0,03 |
| hsa-miR-671-3p | 0 | 0,00 | 1 | 0,00 | 0 | 0,00 |
| hsa-miR-671-5p | 16 | 0,05 | 2 | 0,01 | 7 | 0,02 |
| hsa-miR-675 | 5 | 0,02 | 0 | 0,00 | 0 | 0,00 |
| hsa-miR-675b | 0 | 0,00 | 2 | 0,01 | 0 | 0,00 |
| hsa-miR-7 | 8 | 0,03 | 10 | 0,03 | 12 | 0,03 |
| hsa-miR-708 | 27 | 0,08 | 2 | 0,01 | 22 | 0,05 |
| hsa-miR-708* | 1 | 0,00 | 0 | 0,00 | 0 | 0,00 |
| hsa-miR-7-1* | 13 | 0,04 | 19 | 0,06 | 11 | 0,02 |
| hsa-miR-720 | 3 | 0,01 | 0 | 0,00 | 15 | 0,03 |
| hsa-miR-744 | 8 | 0,03 | 8 | 0,03 | 10 | 0,02 |
| hsa-miR-766 | 9 | 0,03 | 4 | 0,01 | 9 | 0,02 |
| hsa-miR-768-3p | 8 | 0,03 | 0 | 0,00 | 25 | 0,06 |
| hsa-miR-768-5p | 0 | 0,00 | 0 | 0,00 | 4 | 0,01 |
| hsa-miR-769-5p | 6 | 0,02 | 1 | 0,00 | 3 | 0,01 |
| hsa-miR-770-5p | 0 | 0,00 | 0 | 0,00 | 1 | 0,00 |
| hsa-miR-874 | 6 | 0,02 | 1 | 0,00 | 9 | 0,02 |
| hsa-miR-874 5p | 1 | 0,00 | 0 | 0,00 | 4 | 0,01 |
| hsa-miR-886-5p | 0 | 0,00 | 2 | 0,01 | 1 | 0,00 |
| hsa-miR-875-5p | 3 | 0,01 | 0 | 0,00 | 0 | 0,00 |
| hsa-miR-886-5p | 1 | 0,00 | 0 | 0,00 | 0 | 0,00 |
| hsa-miR-887 | 13 | 0,04 | 1 | 0,00 | 3 | 0,01 |
| hsa-miR-888 | 1 | 0,00 | 0 | 0,00 | 0 | 0,00 |
| hsa-miR-889 | 4 | 0,01 | 0 | 0,00 | 1 | 0,00 |
| hsa-miR-891a | 3 | 0,01 | 0 | 0,00 | 0 | 0,00 |
| hsa-miR-9 | 2 | 0,01 | 0 | 0,00 | 3 | 0,01 |
| hsa-miR-9* | 3 | 0,01 | 6 | 0,02 | 3 | 0,01 |
| hsa-miR-92a | 226 | 0,71 | 298 | 1,00 | 405 | 0,91 |
| hsa-miR-92a-1* | 0 | 0,00 | 0 | 0,00 | 1 | 0,00 |
| hsa-miR-92b | 0 | 0,00 | 0 | 0,00 | 23 | 0,05 |
| hsa-miR-93 | 197 | 0,62 | 182 | 0,61 | 152 | 0,34 |
| hsa-miR-93* | 2 | 0,01 | 10 | 0,03 | 2 | 0,00 |
| hsa-miR-936 | 12 | 0,04 | 0 | 0,00 | 0 | 0,00 |
| hsa-miR-940 | 0 | 0,00 | 2 | 0,01 | 0 | 0,00 |
| hsa-miR-941 | 0 | 0,00 | 1 | 0,00 | 0 | 0,00 |
| hsa-miR-942 | 5 | 0,02 | 2 | 0,01 | 3 | 0,01 |
| hsa-miR-944 | 0 | 0,00 | 0 | 0,00 | 1 | 0,00 |
| hsa-miR-95 | 2 | 0,01 | 3 | 0,01 | 5 | 0,01 |
| hsa-miR-96 | 19 | 0,06 | 4 | 0,01 | 3 | 0,01 |
| hsa-miR-96* | 2 | 0,01 | 0 | 0,00 | 0 | 0,00 |
| hsa-miR-98 | 3 | 0,01 | 4 | 0,01 | 2 | 0,00 |
| hsa-miR-98-3p | 1 | 0,00 | 1 | 0,00 | 0 | 0,00 |
| hsa-miR-99a | 78 | 0,24 | 1 | 0,00 | 31 | 0,07 |
| hsa-miR-99a* | 7 | 0,02 | 1 | 0,00 | 2 | 0,00 |
| hsa-miR-99b | 9 | 0,03 | 7 | 0,02 | 14 | 0,03 |
| hsa-miR-99b* | 1 | 0,00 | 0 | 0,00 | 0 | 0,00 |
| pot.new-miR-42 | 0 | 0,00 | 2 | 0,01 | 0 | 0,00 |
| pot.new-miR-27 | 0 | 0,00 | 2 | 0,01 | 0 | 0,00 |
| pot.new-miR-34 | 0 | 0,00 | 2 | 0,01 | 0 | 0,00 |
