# Supplementary material for: MicroRNA Profiling of Epstein-Barr Virus-Associated NK/T-Cell Lymphomas by Deep Sequencing
Source: PLoS One. 2012 Aug 3;7(8):e42193. doi: 10.1371/journal.pone.0042193 (PMC3411711; doi:10.1371/journal.pone.0042193)
Supplement: Table S5 — New miRNAs from unknown precursors identified from EBV-negative T-cell lymphoma. (DOCX) [file pone.0042193.s011.docx]

**Supporting Table S5**

| **name** | **miRNA-precursor** | **dG** | **genomic localisation** | **conservation** |
| --- | --- | --- | --- | --- |
|  |  |  |  |  |
| miR pot.27 |  | -33,9 | 10q11.23 | ptr |
| miR pot.34 |  | -39,5 | 20q11.23 | ptr |
| miR pot.42 |  | -41,8 | 10q24.1 | ptr |
